# An empirical evaluation of a novel domain-specific language – modelling vehicle routing problems with Athos

**DOI:** 10.1007/s10664-022-10210-w

**Published:** 2022-09-23

**Authors:** Benjamin Hoffmann, Neil Urquhart, Kevin Chalmers, Michael Guckert

**Affiliations:** 1 Kompetenzzentrum für Informationstechnologie, Technische Hochschule Mittelhessen, Friedberg, Germany; 2 School of Computing, Edinburgh Napier University, Edinburgh, Scotland; 3 School of Arts, University of Roehampton, London, England

**Keywords:** Domain-specific languages, General-purpose language, Empirical evaluation, Vehicle routing problem

## Abstract

Domain-specific languages (DSLs) are a popular approach among software engineers who demand for a tailored development interface. A DSL-based approach allows to encapsulate the intricacies of the target platform in transformations that turn DSL models into executable software code. Often, DSLs are even claimed to reduce development complexity to a level that allows them to be successfully applied by domain-experts with limited programming knowledge. Recent research has produced some scientifically backed insights on the benefits and limitations of DSLs. Further empirical studies are required to build a sufficient body of knowledge from which support for different claims related to DSLs can be derived. In this research study, we adopt current DSL evaluation approaches to investigate potential gains in terms of effectiveness and efficiency, through the application of our DSL *Athos*, a language developed for the domain of traffic and transportation simulation and optimisation. We compare Athos to the alternative of using an application library defined within a general-purpose language (GPL). We specified two sets of structurally identical tasks from the domain of vehicle routing problems and asked study groups with differing levels of programming knowledge to solve the tasks with the two approaches. The results show that inexperienced participants achieved considerable gains in effectiveness and efficiency with the usage of Athos DSL. Though hinting at Athos being the more efficient approach, the results were less distinct for more experienced programmers. The vast majority of participants stated to prefer working with Athos over the usage of the presented GPL’s API.

**Supplementary Information:**

The online version contains supplementary material available at 10.1007/s10664-022-10210-whttps://doi.org/10.1007/s10664-022-10210-w.

## Introduction

### Problem Statement

A domain-specific language (DSL) is a programming language that is tailored towards the requirements of a specific problem domain (Mernik et al. 2005). The potential advantages of an appropriately designed DSL include the abstraction of technical platform details (Iung et al. 2020), facilitated program comprehension (do Nascimento et al. 2012), and the elimination of error-prone, repetitive tasks (Hermans et al. 2009). These advantages are claimed to result in an increased productivity (Barišić et al. 2012a). A model written in a DSL is usually processed by a generator that relies on templates created by software engineering experts. This way, the generated code follows best practice implementations and reflects deep software engineering expertise.

The reduction in the level of required software development skills potentially allows domain experts to develop applications for their domain. Using the DSL means to create a model and not to write code. This raises the questions whether modelling through a DSL is more effective than coding and whether the results are of comparable quality. Efficiency and ease of use are crucial quality criteria of a DSL. In this paper, we address this question for the domain of vehicle routing problems (VRPs) and their solution. Our DSL *Athos* provides a declarative language in which problems together with algorithmic solutions can be described and solved. The Athos generator transforms models into executable code for multi-agent platforms, e.g. NetLogo (Tisue and Wilensky 2004).

Until recently, the aforementioned claims on the benefits brought about by DSLs have often received only anecdotal affirmation (Kosar et al. 2012; Barišić et al. 2012b). Quantitative language evaluations have mostly been neglected (Barišić et al. 2012a) or conducted using controvertible approaches (Challenger et al. 2016). In their systematic mapping study, Kosar et al. (2016) found that software language engineers (SLEs) only very scarcely report on language evaluation. This is all the more disconcerting given that the development of DSLs requires a considerable initial effort. To justify this investment, it is crucial that the final product is not unthinkingly put to use. Instead, the added value of a DSL as well as its potential for further improvement should be brought to light via empirical evaluation (Barišić et al. 2014).

Although recently there has been an increased interest in the field of scientific DSL evaluation (Kosar et al. 2018), it is still in its early stages. Additional holistic evaluations from various fields are required in order to support researchers and developers likewise in the adoption of approaches based on DSLs (Challenger et al. 2016). Kosar et al. (2018) stress the importance of researchers publishing their results on DSL evaluations in order to create a substantial body of literature on the subject. Barišić et al. (2014) point out that a single evaluation study is only of limited informative value whereas real confidence can only be gained from several evaluation studies with consistent findings. The authors thus emphasise the importance of a solid body of knowledge from which sound and valuable conclusions can be drawn.

### Research Objectives

In order to facilitate the integration of software engineering studies that employ controlled experiments into a coherent body of knowledge, Jedlitschka et al. (2008) propose the usage of the goal template developed as part of the goal/question/metric method (Basili 1994; van Solingen et al. 1999). Usage of this template is supposed to ensure a concise provision of overview on the studies objects, purpose, quality focus as well as its perspective and context. The research objectives of this study can thus be summarised as follows:**Analyze** Athos and JSprit**for the purpose of** their evaluation and comparison**with respect to** their respective effectiveness, efficiency and user satisfaction**from the viewpoint of** experts from the domain of vehicle-routing and software developers**in the context of** the Operations Management (OM) lecture at the Technische Hochschule Mittelhessen (THM) campus in Friedberg and the Model-Driven Software Development lecture at the Technische Hochschule Mittelhessen campus in Wetzlar.

The study presented in this paper compared two languages for modelling vehicle routing problems with time windows (VRPTWs) in terms of how effectively and efficiently users could comprehend and create VRPTW models and how satisfied they were with the respective approach. According to the quality-in-use model defined in ISO/IEC 25010 (20110), effectiveness, efficiency and satisfaction are those characteristics that define the usability of a software component. In other words, the presented study evaluates and compares the usability of two VRPTW modelling approaches. For this, students from two different study courses were selected to form the study’s sample population representing domain experts and software developers as potential target users.

### Context

Two studies were conducted among students of two different study courses offered at two different campuses of Technische Hochschule Hessen (THM). The first study mainly featured participants who were at the beginning of their programming careers. These students were enrolled in a study course on information systems offered at the Friedberg campus. Most participants were in their second semester of study. The second study was conducted among participants with more advanced programming experience gained in real-world industrial projects. These participants were enrolled in a co-operative course on software engineering offered at the campus for co-operative study courses located in Wetzlar. These participants were in their fourth semester and actively taking part in software development projects of their employing company. Participants were not directly rewarded for participation in the study. An indirect reward was announced as a task on both of the compared languages was agreed to be part of the final exam and participation in the study was to serve as a training session towards that task.

In the next section, we discuss related DSL evaluation studies. After providing a general introduction to Athos and its tooling in Section 3, we present the details of our evaluation study in Section 4. In Section 5, we analyse the results of the study and consider the potential threats to validity in Section 6. Finally, in Section 7, we summarise the main findings and conclude this paper.

## Related Work

### Studies Comparing DSLs and Application Libraries

Kosar et al. (2010) showed that DSLs can significantly improve program/model understanding compared to the adoption of application libraries used within general purpose languages (GPLs). The authors designed two questionnaires with tasks of comparable complexity: the tasks of the first questionnaire had to be solved using a DSL (XAML), and for the tasks of the second questionnaire an application library of a GPL (C# forms) had to be used. The tasks of both questionnaires stemmed from the domain of graphical user interface development. They were designed in a way that tested how efficiently study participants could a) learn the notation, b) perceive the programs, and c) evolve given programs/models of the respective approach. The authors conducted their study with 36 participants, all of whom received an introduction to the problem domain and training in both approaches. After participants answered the questionnaires, the authors calculated the participants’ success rate for all tasks. The authors then were able to statistically prove that the application of a DSL for GUI development has a beneficial effect on the success rate. The authors hence concluded that DSLs have the potential to facilitate program understanding.

Kosar et al. (2012) then expanded their original experiment to a family of structurally similar experiments. The family consisted of three experiments each situated in a distinct domain (feature diagrams, graph description and GUIs). In each experiment a DSL was compared to either a GPL application library or an application programming interface (API) for a GPL. In their study, the authors researched whether DSLs positively affected program comprehension among participants.

In this study the authors took a *within-subjects* approach so that for each experiment two distinct groups were formed. The first group started with the DSL and then the GPL; the second group used the inverse order. The authors aggregated the results obtained from both group and applied a parameterless test for dependent samples (Wilcoxon signed-rank test).

The obtained results show that in each of experiment usage of the DSL led to significantly improved results. In all three explored domains, the results give proof to the claim that DSLs possess the potential to enhance the performance of developers. It is to be noted that a generalisation of these findings to other domains is difficult if not impossible. In other domains the observed outcome might be markedly different. Therefore, it is of high importance to conduct further empirical evaluation studies.

Kosar et al. (2018) performed a modified replication of their family of experiments. The most important modification of the original study was the replacement of pen and paper by integrated development environment (IDE). Instead of having to manually write down their answers, participants were allowed to use IDEs to solve the study tasks. Another modification was that the study design was changed from *within-subjects* to *between-subjects*, i.e. participants no longer solved both the DSL *and* GPL tasks but either the one or the other. To gain some further insight into how participants used the IDEs, they also asked participants to state which category of tools they used for each task.

The replication study evaluated languages from the same three domains as the original (feature diagrams, graph description, and GUIs). For each domain participants had to solve a set of tasks that involved the respective DSL or the GPL. As was already mentioned, in the replication study, the design of the study was modified so that no participant answered the questions of the same domain with both the DSL and the GPL. The dependent variables of the study again were *effectiveness* and *efficiency*.

The results suggest that IDE support positively affected the effectiveness and efficiency of both DSL and GPL users. Other than that, the results are consistent with those of the original study. In all three domains DSL users achieved significantly better effectiveness results than GPL users. At the same time, DSL users were also significantly more efficient. An in-depth analysis, however, reveals that questions from the *evolve* category are a major reason for the superior results of DSL users. In the other two question categories, results were less distinct. This is where the replication differs from the original study (where DSL users achieved significantly better results throughout all question categories in all three domains). The authors deem the IDE support responsible for this. They consider it likely that IDEs benefit GPLs more than DSLs.

All three experimental studies of Kosar et al. provide evidence that DSLs are a suitable approach to support software developers in minimising programming / modelling errors while at the same time helping them to become more efficient. Moreover, the replication study also provides some interesting findings related to the deployment of IDEs. Though we agree that IDE support of DSLs is a topic that merits further investigation, for our study, we decided to not allow IDE usage. It is to be noted that this decision poses a possible threat to the external validity of this study (see Section 6.4). We still went with this decision, in order to be able to focus on Athos’ *abstract* and *concrete syntax* and on how well these compare to those of a selected baseline approach. Allowing the application of an IDE would introduce the level of IDE support as a confounding variable.

Johanson and Hasselbring (2017) present a similar study in which they compare their Sprat Ecosystem DSL to an application library. The authors tailored their DSL for the domain of marine ecosystems.Two important use cases covered by the DSL are parametrization and data recording. Parametrization refers to the specification of simulation scenarios. Data recording refers to the specification of the data to be produced in the course of a simulation run (data recording). For both use cases a task was defined. Participants approached both tasks with both languages in a randomised order (within-subjects design).

The population of the presented study consisted of experts from the domain of marine ecosystems from different research groups located in various countries. The study focused on how effective and efficient participants solved two different tasks with the compared approaches. The authors also investigated how satisfied users were with both approaches in terms of various characteristics (e.g., level of abstraction). Finally, the authors investigated whether participants were able to modify (maintain) the language itself via additions to the languages main configuration file.

The study shows that with the DSL participants produced substantially more correct results (on average 61% correctness increase) in a reduced period of time (on average the required time per task was reduced by 31%). The study also provides data that clearly show that participants preferred usage of the DSL to the application of the baseline GPL. The authors also provide evidence which shows that even though participants had only scarce knowledge of the Java language used in the configuration file, an overwhelming portion was capable of performing the required language modification.

There are some important differences between the Sprat DSL evaluation study and the one presented in this article. Most obviously, the evaluated languages target different application domains: Sprat is a DSL for the domain of marine ecosystem simulation whereas Athos is a language for the domain of traffic and vehicle routing problems. However, both languages are similar in that they enable users to specify the entities and parameters of a complex system in order to generate an executable computer simulation. Another important difference is in the studies’ population: In the Sprat evaluation, the population consisted entirely of actual domain experts. In our study, on the other hand, students were invited to participate. Therefore, the results of the Sprat evaluation study are more generalisable to the intended population of language users. However, we believe that our selection of students that we invited to participate ensures that our study’s population represents the targeted users of our DSL reasonably well.

The advantage of asking students for participation shows in the respective population size: in the Sprat evaluation 36 samples were obtained whereas our study produced 101 samples. In terms of quantity, there is also an important difference in the effort participants had to take to solve all study tasks correctly. The Sprat evaluation consisted of two open-ended tasks that could be answered in around 13 minutes. Our study features 15 different tasks – both open ended and multiple choice – that took most participants more than 70 minutes (when facing the tasks for the first time). It thus could be argued that our study is more realistic in terms of including the effects of mental exhaustion which occur during the specification of real-world simulations.

We decided to use the studies of Kosar et al. as a template for our study. The approach of using an appropriate application library as a benchmark to evaluate the benefit of a given DSL holds merit. Especially in cases where no alternative DSL to the one to be evaluated exists, the next best alternative is to compare against an application library of a GPL. We firmly believe that the presented approach should be applied in several other domains in order to evaluate DSLs from these domains as existing study results from different domains obtained with different languages are difficult to generalise. We therefore decided to adopt the approach of Kosar et al. for an evaluation of our DSL that targets the domain of VRPTWs. At this point, it is important to highlight the differences in our approach and the one presented by Kosar et al.: First of all, it is highly likely that the order in which participants answer two questionnaires consisting of similar tasks has an effect on the outcome. It must be assumed that participants learn from the first questionnaire and thus understand and solve the tasks for the second approach more easily. Kosar et al. addressed this issue by conducting two surveys with two different study groups that answered the two questionnaires in reverse order. However, in their presented results, they merged the results from both groups. Without differentiating the results of both groups, it is not possible to prevent learning effects from affecting the results. For this reason, we decided to compare the results of our DSL and the GPL when both are used as a first approach to solve the tasks, and also when both are used as a second approach (more details on this are provided in Section 4). Another important aspect in which this study extends the work of Kosar et al. is through the investigation as to whether the effect of using a DSL is different for participants who have only scarce GPL experience from participants with a deeper understanding of GPL programming. Hence, even though we consider the study of Kosar et al. an important and valuable contribution to DSL evaluation, we believe that our study allows for an even deeper insight into how to interpret the obtained results.

### Similar DSL Evaluation Studies

De Sousa and da Silva (2018) conducted a usability evaluation on DSL3S, a DSL that seeks to facilitate the development of spatial simulations by application of model-driven development (MDD) techniques. The language was implemented as a unified modelling language (UML) profile that introduces stereotypes for core constructs from the domain of spatial simulations such as spatial variables, animats and operations. The language is supported by a tool stack that supports the creation, transformation and execution of models. For their language evaluation, the authors created two artefacts: The first artefact was a step-by-step guide that comprised all the steps beginning at the setup of the necessary tools and a first project to the definition, transformation and execution of a predator-prey model. The second artefact was a questionnaire that recorded participants impressions on the DSL itself, the tools supporting the DSL, and on the MDD approach in general. For each of these three areas the questionnaire featured four questions. Each question asked to rate a specific aspect within the respective area by means of a five-point Likert scale. In addition to these questionnaires, the authors also recorded the profiles of the participants (e.g. profession, area of expertise, or experience with spatial simulations) and how far participants went in the step-by-step guide within a time frame of 50 minutes. The authors obtained somewhat mixed results with a slightly positive tendency in the language area. The ease of learning of the DSL was highly appreciated by participants and the supporting tools of the language were also highly marked. Especially the code generation aspect received almost only prime ratings. Participants appeared to take a rather guarded stance toward MDD in general. Only in the question of whether approaches like DSL3S possess the potential to improve communication to stakeholders, participants clearly gave mostly positive feedback.

De Sousa and da Silva also found significant evidence for biased answers to two of the questions: The ratings for the question on whether DSL3S or MDD in general could serve as a basis for a new standard language for simulation specification showed significant divergence between participants who claimed to be trained in computer science and those who did not. Trained participants seemed to be more appreciative of DSLs as a new standard than participants without training in computer science. This question also showed significant divergence when dividing participants depending on whether they had prior experience in simulations or not. Between these two sub-groups the ratings on the general development process also showed significant deviations. Participants with simulation experience consistently gave positive ratings while those without prior experience gave more diverse ratings. From their findings, the authors conclude that participants appreciated DSL3S, especially the ease with which it can be learned. On the other hand, participants remained cautious towards the general MDD approach. Here, especially two types of participants appeared to be negatively biased towards the MDD approach: participants without training in computer science as well as participants experienced in simulation. On the other hand, it was the group of participants with simulation experience who uniformly appreciated the general development process. The authors thus assume that the scepticism of these participants might be more based on a subjective feeling than on actual problems encountered in the development process.

There are several differences between the evaluation methodology applied by de Sousa and da Silva and the one applied in the study presented in this paper. Firstly, in their study, participants do only get to work with one language. Thus, especially participants who do not have prior experience in the creation of computer simulations have no means to place the evaluated approach into context. This does not necessarily render the ratings of this sub-group of participants less valuable, but it must be kept in mind upon evaluation of the results. In their study, the group of participants without prior knowledge in simulation were less appreciative of the adopted MDD approach than those participants experienced in this field. The diversity in the ratings of this subgroup might be due to the fact that these participants lack the knowledge of an alternative approach and thus had to base their ratings on mere gut instinct. Secondly, their approach does not require participants to use the evaluated language in order to develop a solution for a given task. Instead, participants are only supposed to follow instructions of a step-by-step guide. This is problematic for two reasons: Firstly, the outcome of the language evaluation is largely dependent on the quality of the guide handed to participants. Secondly, most developers have made the first-hand experience with technologies that appeared promising when following an introductory tutorial but turned out to be less helpful when applied without direct guidance. Finally, another important difference between their study and the study presented in this paper is the fact that the evaluation of DSL3S is by and large of qualitative nature. Except for one metric that tracked the progress of participants made within 50 minutes, all results were based on subjective impressions of participants. This might pose a threat to validity since study participants could feel inclined to support study conductors by generally giving more favourable results.

Ewais and de Troyer (2014) conducted a pilot evaluation study on three related graphical DSLs from the domain of adaptive three-dimensional virtual learning environments (VLEs): The first language, named *pedagogical model language (PML)* is concerned with the pedagogical structure of the VLE. Its main syntactical elements are pedagogical relationship types (PRT) and *pedagogical update rules (PURs)*. PRTs are applied to define relations between different learning concepts (e.g. one learning concept has to be mastered before another can be accessed). PURs are rules that when triggered update the learner’s profile given that pre-defined conditions hold. The second language of the evaluation was the *Adaptive StoyLine Language (ASLL)*. Its purpose is the definition of a narrative arc (or learning path) for VLEs. For this, it allows to structure learning concepts by the definition of *topics.* Topics can be connected via so-called *storyline adaptation rules* that are used to define the circumstances under which a learner can advance to a new topic. *Adaptive topic language (ATL)* is the third language evaluated in the study. In contrast to ASLL, it is used inside a single topic in order to define how the content of the given topic adapts to a learners behaviour or status. For this, it allows to define adaptation rules. These rules define events of a source learning concept as triggers for the evaluation of conditions. They also define what adaptation is to be performed on a target learning concept in case the checked condition holds.

The study was conducted among 14 participants working at the same computer science department as the authors. The study was composed of three main steps: In the first step, they introduced participants to the three DSLs. In step two, participants were supposed to use the DSLs to author an adaptive 3D VLE on the solar system. It is to be noted that participants had to perform this task using pen and paper while receiving support on the languages from an instructor. The time for completing the definition of the VLE was taken. In the third step, participants answered a questionnaire that was divided into six categories (demographics, specification of VLEs, and four more questionnaires/feedback instruments from the literature).

Analysing the usability of their language, the authors claim that they received good ratings for all questions on five out of seven general ergonomic principles defined in ISO 9241/10 (five questions for each of these principles were defined by Prümper (1999), though the paper does not state whether all of these questions were used). Questions regarding the principle of “suitability for learning” are reported to be neutrally rated. For the analysis of the acceptability of the three DSLs, the authors subsumed 11 questions under this term. They report that 7 of these questions received good ratings, whereas 4 were rated neutrally. The questionnaire also posed open questions that allowed participants to give feedback on what they liked, what they disliked and suggestions on how to improve the DSLs. Interestingly, the majority of the participants appreciated that three DSLs had to be applied for the definition of a VLE. Users disliked that the approach distinguished between *adaptive storyline* and *adaptive topic*. Most recommendations concerned the need for an editing tool.

Other than the time it took participants to complete the task, the study does not feature any metrics that give insight on how well participants performed in the second step of the study. Instead, the study is almost entirely made up of questions that ask for participants’ opinion. This is a major difference when comparing their study to the study presented in this article. While our study also contains a number of questions that investigate the impression Athos left with participants, most of the questions of our study are tasks designed to gain insight on the results participants create by application of the DSL. Another difference is the fact that Ewais and de Troyer have not presented their participants an alternative approach to solve the task at hand which might make it harder for participants to correctly assess the quality of the evaluated DSL. It also must be noted that the authors do neither provide an overview of all the questions asked in their questionnaire, nor do they elaborate on how they calculated whether a complete question was rated as “poor”, “neutral”, or “good”.

### Additional DSL Evaluation Studies

Tekinerdogan and Arkin (2019) present ParDSL - a framework of four DSLs that support language users in mapping parallel algorithms to target parallel computing platforms. The authors designed each of the four languages for one specific activity involved in this mapping process. The framework thus comprises each a DSL that allows to model a) the physical target platform, b) the decomposition of the parallel algorithm to be mapped, c) the logical structure of the target platform, and d) the transformations into executable code. The authors evaluate their ParDSL framework by discussing it in terms of several quality characteristics defined in the FQAD DSL evaluation framework (Kahraman and Bilgen 2015).

The presented evaluation is, however, not conducted based on a controlled experiment. The authors explain that the number of trained language users would not suffice to conduct an empirical evaluation study from which a statistically valid result could be obtained. The authors therefore provide a comprehensive discussion of their DSL framework in terms of several FQAD quality characteristics. For example, the authors argue that ParDSL is a *functionally suitable* language because it fulfills the functionality that was required in their presented examples. The authors base the usability evaluation of their framework on a set of informal interviews that were conducted among senior engineers and senior faculty members. In these interviews, the framework was lauded for its expressiveness in relation to its simplicity. Especially the separation of the four main activities of the mapping process was positively recognised.

Cordasco et al. (2021) conducted a performance evaluation of their Fly DSL. Fly was developed in order to enable experts from different domains to develop parallelised applications without being experts in distributed cloud systems. The language thus allows users to model parallel data processing functions (or work-flows) on several different cloud services while making the necessary technical intricacies transparent. In the presented performance evaluation, the authors apply their language to several instances of the word count problem, a well-known benchmark problem in the parallel computing domain. The authors create a Fly program for the benchmark problem and vary several parameters (file size, number of files) to gain insight into how performance and cost aspects compare when using different sequential and parallel execution settings. In addition to this benchmark problem, the authors also apply their language to model and execute two algorithms from two distinct scientific use cases. As a baseline approach, the authors used a single-threaded Java execution and analysed the differences in performance and cost.

While Fly is similar to Athos in that it is tailored for domain experts with little to no knowledge of computational technicalities and while both Athos and Fly were developed with the Xtext^1^ framework, the presented study is very different from the one presented in this article. First of all, Fly is a DSL targeted at a specific technical domain (parallelised, cloud-based computing) whereas Athos targets a specific application domain (vehicle routing and traffic). Another very important difference is that the presented study seeks to evaluate how well the generated code performs on different problem instances and on different target architectures. By contrast, in our study execution performance is not relevant. The controlled experiment presented here is to provide insight if and to what extend Athos enhances domain experts’ effectiveness, efficiency as well as their tool satisfaction.

### Frameworks for DSL Evaluation and Example Studies

Challenger et al. (2016) introduce a multi-agent systems (MASs) DSL evaluation framework. The framework consists of a *language dimension*, an *execution dimension* and a *quality dimension*. The first two dimensions evaluate the DSLs by application of quantitative criteria while the latter takes a qualitative approach. Each dimension is further split into two *sub-dimensions* that define *evaluation criteria* for the respective aspect of the DSL. As an example, the language dimension is divided into the *language elements* and *model transformations* sub-dimensions. Evaluation criteria for the *language elements* sub-dimension are the number of *abstract and concrete syntax* elements and the *number of constraints (static semantics)*. Criteria of the *model transformation* sub-dimension is the number of model-to-model (M2M) and model-to-text (M2T) transformations.

To demonstrate the application of the framework, the framework is used to evaluate a language called *SEA_ML*, a language developed by the authors. Applying the criteria of the *language dimension*, the authors state the number of meta-model elements, attributes, etc. and note that these numbers can also be determined for any other MAS related DSL in order to compare it to SEA_ML. They argue that the language with more elements is likely to be more expressive and allows for more detailed modelling.

For the evaluation of the other two dimensions whose criteria rely on an actual application of the language, the authors conducted a multi-case study. The study featured four scenarios from different application domains. Each scenario required the development of a MAS for a particular domain. Members of the study group were asked to apply SEA_ML for domain analysis and domain modelling as well as implementation, testing and maintenance of the respective MAS. Members of the control group were asked to perform these steps with a combination of general-purpose modelling notations (e.g. UML) and agent-development methodologies (e.g. Tropos) together with manual code development for a multi-agent platform (JADEX or JACK).

As a quantitative result, the authors state that on average around 80% of the final product could be generated while the remaining 20% required manual implementation. They claim that this substantially reduces the complexity of MAS development. Another quantitative result is that SEA_ML considerably accelerates the development process: study group members required only an average of 3.5 hours per case study – opposed to an average of 6.25 hours required by control group members. The most notable efficiency gain occurred in the implementation phase: study group members required an average of 37 minutes for this phase. For control group members, the average time spent for implementation was nearly four times as much. The study group also outperformed the control group in the testing and the maintenance phases. In both phases they required only around half the time (testing: 17 vs. 34 minutes, maintenance: 26 vs. 50 minutes).

With their framework and the presented study, Challenger et al. have made important contributions to the field of DSL development. They provide criteria for the assessment of a wide range of DSL components and present an example of how to obtain the data for these criteria. The main purpose of these criteria is an assessment of the expressiveness of a DSL as well as an assessment of the added value in terms of efficiency. Although the presented approach is to be considered one of the most sophisticated approaches in the field of DSL evaluation, it is important to note that it was mainly designed for graphical DSLs. The framework lacks some criteria, that might be especially important when dealing with textual DSLs. For example, the framework seems to neglect the fact that language users often introduce syntactical and semantic mistakes when creating DSL models. Although it can be argued that graphical DSLs are less prone to these types of mistakes, the framework still might benefit from the introduction of additional criteria that allow further insight on the number or percentage of mistakes made by new language users. The framework also seems to overlook the importance of the *learnability* and *perceivability* of the evaluated language. A deeper insight on both aspects might be obtainable by an analysis of the number of modelling mistakes made by language users.

Another framework designed to guide DSL evaluation studies is the Usa-DSL evaluation framework^2^ (Poltronieri et al. 2018). Following the works of Barišić et al. (2012b, 2014), the framework treats DSLs as interfaces enabling human-computer interaction. Usa-DSL focuses on the usability aspect of a DSL. Usability is defined within the context of the *quality in use* model (ISO/IEC 25010 2011) as being composed of three characteristics: effectiveness, efficiency and satisfaction. Usa-DSL defines abstract guidelines that define the flow of activities and artefacts in the evaluation process (Poltronieri et al. 2021). The framework does not prescribe the exact implementation of specific activities in the evaluation process. Instead, the framework provides adequate suggestions on the order and embodiment of evaluation activities so that it is left to the experiment conductor, to compose a concrete evaluation process from the various possible paths. In addition to the work of Kosar et al., we also considered the Usa-DSL framework in the planning, execution, analysis and reporting of this study.

## Athos – a DSL for Vehicle Routing and Traffic Simulations

### Tooling and the Underlying Architecture of the Language

Athos was implemented using the Xtext language generation framework^3^ (version 2.12.0). Xtext allows to create language-supporting tools like editors, compilers, validators and generators as Eclipse plug-ins. Hence, Athos can be installed into current Eclipse IDEs as a plug-in from our update site. When Athos is installed, it can be used from within the Eclipse IDE to define traffic and transport simulations and optimisation models.

**Fig. 1:**
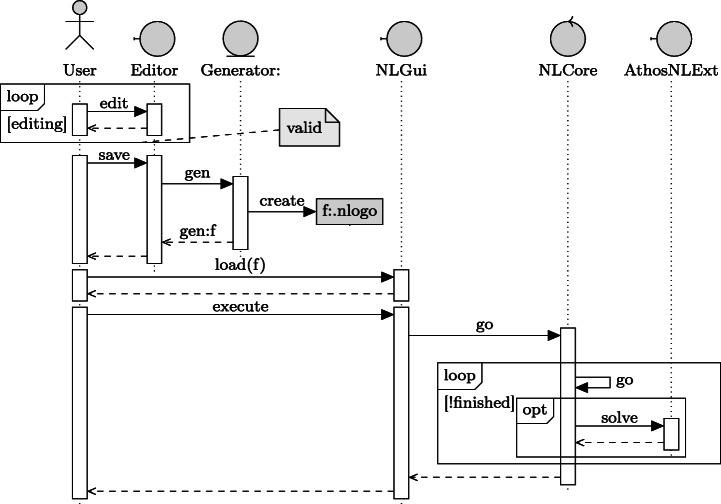
Interaction of entities in using Athos

Figure 1 provides an overview of the general workflow and the internal processes inside the Athos framework: users can create textual models using the sophisticated Athos editor which (due to the underlying Xtext framework in combination with appropriate modifications and extensions of the framework-generated code) offers a series of helpful features like syntax-highlighting, code completion and validation and quick-fix mechanisms. Provided that the current state of the Athos model in the editor is valid (i.e. there are no syntactical errors in the code and all validation checks are passed), saving the model triggers the generator. The generator was implemented with the Xtend^4^ language. It produces a model file for the NetLogo^5^ platform from the Athos model. The generated NetLogo model can then be loaded and run by the NetLogo platform. Depending on the Athos model, the NetLogo platform may need to solve various VRPs. For these problems, a NetLogo extension providing heuristic optimisation algorithms was developed. If necessary, the platform can leverage this extension to obtain near-optimal solutions which then are transformed into agent-behaviour within the simulation. Athos also allows to specify metrics, i.e. mathematical expressions that include events or data of interest. These metrics are also transformed appropriately to NetLogo code so that the NetLogo platform tracks the defined metrics during the simulation run and saves them to a result file (Hoffmann et al. 2019).

With the presented process and its components it is possible to create complex NetLogo simulations from Athos models that in general only need a fraction of the lines of code (Hoffmann et al. 2018). Though currently, we have chosen NetLogo as our main target platform, it is also possible to change the target-platform to different multi-agent simulation frameworks (Hoffmann et al. 2018).

### Athos by Example

In this section, we provide a brief overview on the structure and syntax of Athos programs for VRPTWs (c.f., e.g. Ombuki et al. 2006) by presenting and discussing a simple example. The presented example is the one that was used in the Athos and JSprit learning material that was presented to study participants (see Section 4.5).

**Listing 1:**
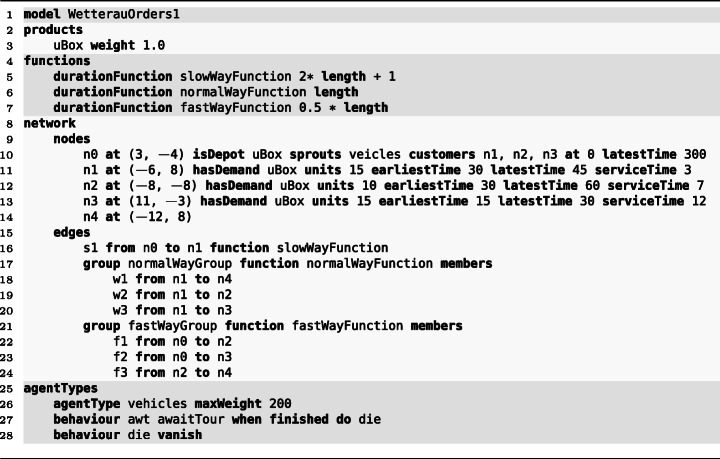
General structure of an Athos Model (alternating colors indicate the different program sections)

Listing 1 is a complete Athos program that models a simple VRPTW instance. Line 1 is the *preamble* where a name for the model (and optionally its spatial dimensions) are specified. Lines 2–3 constitute the *product section*. For classic academic problems like the example at hand, the product often is not of interest. In these cases, one product with an arbitrary name (here *uBox*) and a weight of 1.0 should be modelled. The weight capacity modelled for a vehicle (line 26) then determines how many units of the product the vehicle can transport. It is worth noting that for real-world problems, the possibility of defining several different programs with different weights (and volumes) can facilitate the modelling process for domain experts.

Lines 4–7 represent the *function section*. In this section, *durationFunctions* can be defined. These functions feature a mathematical expression that determine the amount of time it takes a vehicle to traverse an edge to which the respective function is associated. As an example, the definition of the *fastWayFunction* in line 7 says that an edge associated with this function can be traversed in an amount of time^6^ equal to half of the length of the edge.^7^ Though not of interest in this particular scenario, it should be noted that functions can be used to model congestion effects in a network as we have shown in Hoffmann et al. (2018).

The section spanning lines 8–24 is referred to as the *network section*. It consists of two subsections referred to as the *nodes* and the *edges* section. The *nodes section* (lines 9–14) defines all places of the network a vehicle can visit or emerge from: In line 10, a depot named *n*0 located at *x* = 3 and *y* = − 4 is defined. The depot stores and delivers the uBox product and hosts a fleet of vehicles of type *vehicles*. The depot serves customers located at nodes *n*1,*n*2, and *n*3. Vehicles begin their delivery service at time 0 and have to return to the depot by time 300. Lines 11–13 define the customer nodes. For each customer the demanded product together with the demanded quantity is defined.^8^ Within a VRPTW instance each customer has a time window within which the servicing has to commence and a time it takes the vehicle to service the customer. Line 11, for example, states that customer *n*1 has to be serviced in the time interval [30,45] and once the vehicles starts servicing the customer, it takes 3 units of time to complete the servicing procedure. Nodes without any demands serve as navigational nodes of a network.

In the edges section from line 15 to 24 the different roads of the network are defined. In this section, the linking between an edge and a function from the function section takes place. The program also shows two approaches of defining edges in an Athos model: In line 16 a single edge between nodes *n*0 and *n*1 is defined linked with the *slowWayFunction*. The alternative approach is to group edges together which is done twice in the example program. By grouping edges, the associated function has to be stated only once which results in less typing and faster reading of programs. Please note that it is also possible to associate other attributes to edges via this mechanism.

The final part of the presented Athos program is referred to as the *agent section*. Here, the vehicles or more generally agents, i.e. acting entities, are defined. Lines 25–28 define the *vehicles* type with a carrying capacity of 200 weight units. Line 27 states via the *awaitTour* behaviour that the vehicle idles at its depot until it is given a tour of customers to service and vanish from the simulation when the tour is completed. In (Hoffmann et al. 2020), we provide further insight on the different behaviours of vehicles (agents) and how these behaviours are implemented by modelling vehicles as state machines.

**Fig. 2:**
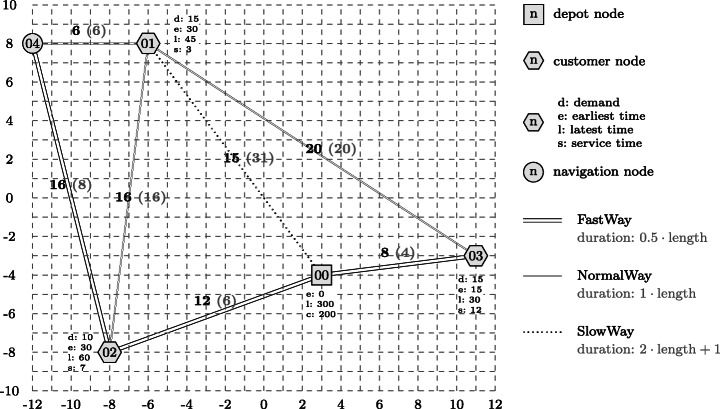
Pictures from the corresponding study questions Q09ATNW and Q09JSNW: The nodes (depot), (customer), (navigation) and edges (roads), (highways) represent elements for which pre-defined code was given in the task. Elements emphasized by bold black colour had to be added to the program by customers

Figure 2 visualises the code of the Athos program. Customers are annotated with their demands, time windows and service times, the depot is annotated with its time window and the capacity of the vehicles stationed at the depot. A similar network representation is created by the NetLogo platform from the generated NetLogo code. Since this visualisation cannot be seen by the modeler until the generated NetLogo simulation is run, we are currently working on an additional plug-in that synchronises with the editor and visualises the code of Athos models and also allows to modify Athos programs by pointing and clicking on the visualised elements.

## Materials and Methods

### General Evaluation Framework and Selection of Study Method

For this study we considered the *phases* and *steps* defined in the Usa-DSL evaluation framework (Poltronieri et al. 2018) and performed the *activities* defined within the framework. The Usa-DSL framework does not imperatively define the exact activity to perform. It guides researchers by providing reasonable suggestions. The exact embodiment of most activities is to be defined by the researcher. For example, *step 4 empirical study method (SE)* of the *planning phase* demands the researcher to *define the empirical study method* but does not demand a specific study method to be embodied.

During the planning phase of this study, in which we decided on the exact activities to perform, it was determined that the most promising and objective approach to gain insight on the qualities of our DSL was to compare it to a baseline DSL. However, to the best of our knowledge, no DSL that would allow for a sensible comparison exists. We then decided to follow the approach taken by Kosar et al. (2010, 2012, 2018) and modify some important aspects.

### Research Questions and Hypothesis

Our experimental study was in large parts inspired by the work of Kosar et al. (2010, 2012, 2018). As a logical consequence, the formulation of the research questions and hypothesis is also based on their work.

1. Does Athos enhance the effectiveness in model comprehension and creation?
2. Does Athos enhance the efficiency in model comprehension and creation?
3. Does Athos enhance the satisfaction of language users?

From these research questions the following three hypothesis were derived:

1. The use of Athos has a positive effect on participants’ effectiveness.
2. The use of Athos has a positive effect on participants’ efficiency.
3. The use of Athos has a positive effect on participants satisfaction.

### Demographic Information and Ethical Considerations

As Athos was designed for experts from the domain of routing optimisation and traffic planning, it was originally intended to have these experts participate in the study. However, it soon became clear that this was impractical for a pilot study since every participant would have had to be introduced to Athos and the application library and it would have been hard to find a date suiting all domain experts so that individual training would have been necessary. In addition, it was noted that most domain experts would not possess enough knowledge of a GPL and hence could not be introduced to an application library in a reasonable amount of time. Therefore, focusing on domain experts as study participants would have led to a very small study population that would then reduce the statistical significance of the discovered results.

For this reason we opted for students from *Technische Hochschule Mittelessen (THM)* enrolled in courses that would ensure a certain level of knowledge in a given GPL. This way, we could focus on introducing participants to the DSL and the baseline application library (and not the underlying GPL). It was decided to perform the study among two different student groups at two different campuses of THM. The first study was to take place at the campus in Friedberg, Germany, among students studying information systems. The study was integrated into a module on operations management which is taken by students in their second semester. The second study was to be conducted among students enrolled in software technology at THM’s co-op campus (StudiumPlus) in Wetzlar, Germany. There, the study was to be integrated in a module on model-driven software development. Please note, that in both study groups students were taught aspects of the study problem relevant to the respective module.

An informed consent form was presented to each participant, along with an explanation of the study. Participants were informed that participation was on a voluntary basis and that all data would be anonymised and thus not be attributable to an individual participant. Ethical clearance was granted by both Edinburgh Napier University and THM.

Initially it was intended to perform the study on the premises of THM, but due to the Covid-19 pandemic it was necessary to perform the study via an online meeting software. The study was designed and carried out using the NoviSurvey online survey construction software. During execution of the study, participants were granted help if they had problems to understand the tasks. Participants were allowed to use notepad and screenshot software in order to solve the tasks.

### Selection of Baseline Application Library and Definition of Problem Domain

In accordance with the study method described by Kosar et al. (2010), it was necessary to find an application library against which Athos was to be compared. With JSprit,^9^ we found an application library for the domain of vehicle-routing optimisation. Before the tasks of the controlled experiment could be defined, it was necessary to look at the capabilities of the two approaches about to be compared. Even though Athos and JSprit have a common core of capabilities, each approach also possesses some features that the other does not offer. In order to devise a set of comparable tasks for each approach, the tasks had to be restricted to features offered by both approaches.

**Fig. 3:**
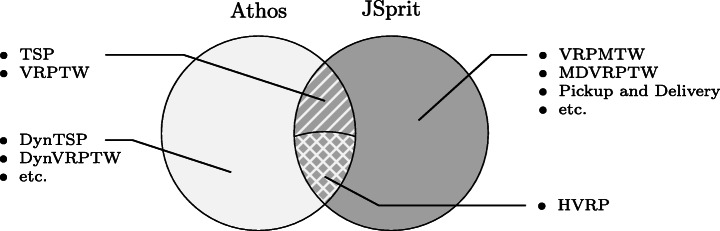
Comparison of the capabilities of Athos and JSprit

Figure 3 illustrates the capabilities/features of both approaches by visualising the set of capabilities of each approach as a Venn-Diagram. Athos models, on the one hand, are automatically transformed into executable simulations in which all actors can mutually influence their respective behaviour (e.g. by slowing down movement speed on a given road so that another actor opts for a longer road which allows faster movement), it can be used to define both *static* and *dynamic* vehicle routing problems (cf., e.g., Pillac et al. 2013).

JSprit, on the other hand, was developed for *static* vehicle routing problems (Mayer et al. 2016; Welch 2017); hence it does not support the specification of problems, in which new actors dynamically enter and alter the original scenario. In contrast to Athos, however, it allows to specify and solve a larger number of static vehicle routing problems, e.g. vehicle routing problems with multiple time windows (VRPMTW), multiple depots (MDVRPTW), or pick-up and delivery problems (PDP), in which a product has to be fetched from one location to be delivered to a different one. The tasks in the controlled experiment were designed so that only functionality in the intersection of JSprit and Athos capabilities were to be solved.

### The Study Protocol

**Fig. 4:**
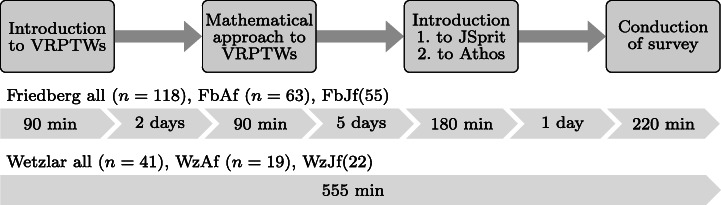
Study conduction protocol

In this section, we describe how we conducted the two studies in Friedberg and Wetzlar. Figure 4 illustrates the performed steps: The rectangles in the upper part of the illustration represent the performed steps in a chronological order. Below these steps, the number of participants in the respective study (Friedberg, and Wetzlar) as well as its groups (e.g. Friedberg, Athos first) is given. In addition, below the information on each study, a timeline shows how much time the respective study group took for the steps and the breaks in between.

In the first step, participants were introduced to the domain of VRPTW. For this, a fictitious case study was developed and presented to the participants. The case study demonstrated the objective function and the constraints of VRPTWs using a scenario in which a grocery retailer chose to introduce a food delivery service. This way, participants learned about the constituting entities of VRPTWs and their relations. In the next step, participants learned about various mathematical aspects like deriving a graph from an incomplete network. In addition, participants were presented a formal mathematical definition of the VRPTW.

Having imparted substantiated domain knowledge to participants, they were introduced to the two approaches compared in the study. Slides were prepared for both approaches that explained how the VRPTW from the case study could be modelled. The first approach presented to participants was the JSprit application followed by Athos as an alternative approach. Extreme care was taken to introduce both approaches with the same due diligence as to not affect the results by inferior learning material or inferior explanations of one of the approaches (also see Section 6).

In both Friedberg and Wetzlar, participants were randomly assigned to the Athos first and JSprit first groups (see next section). This was important since the tasks for both approaches were very similar (see Section 4.7) and thus it was highly likely that the tasks for the second approach might have been easier for participants due to learning effects from the tasks of the first approach. On the other hand, by the time a participant started to solve the tasks of the second approach, exhaustion effects might have started to negatively affect the performance of the participant. In order to be able to compare results free of any learning and exhaustion effects, we decided to split each study into (sub-)groups that applied the approaches in reverse order (see next section). Participants were then given the survey URL from which they could access the survey with an arbitrary browser software. After some final words of explanation regarding the voluntary basis of participation, data protection and the maximum time limit for either approach participants were asked to begin the study. Participants had two example programs at their disposal. Since we had no means of prohibiting usage of additional tools or help, participants were also allowed to use any means they deemed fit to help them in answering the questions.

As depicted by Fig. 4, the Friedberg study consisted of 118 participants. 63 of these participants were in the group that started with the Athos tasks with JSprit as the second approach (group FbAf); 55 participants were in the group that started with the JSprit tasks. The Wetzlar study comprised 41 participants, 19 of which starting with the Athos tasks and 22 beginning with the JSprit questions.

Figure 4 also provides some insight on temporal aspects of the study protocol. In the Friedberg study, the study took place throughout the course of several days with one to several days between the events / steps. The Wetzlar study, due to organisational reasons, had to be conducted within one single day and we were forced to reduce the time for mathematical explanations. It is important to note that this might have an impact on the results of the respective approaches but does not threat the validity of comparisons between the groups of one study since both groups followed the same study protocol.

### Definition of the Survey Structure

**Fig. 5:**
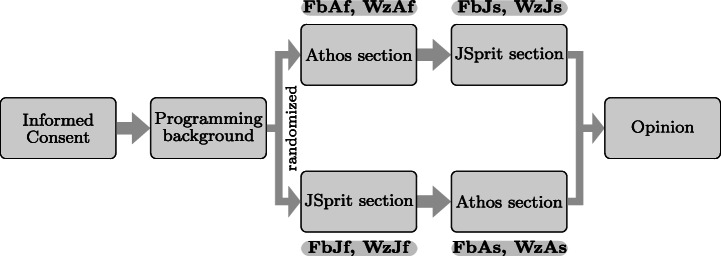
Sequence in which participants answered the five sections of the survey; both studies were split into groups that entered the taks sections for Athos and JSprit in opposite order

Figure 5 depicts the five sections of the survey together with the sequence in which participants entered them. Before participants could enter any other section, they had to consent to the terms of the survey given in the *informed consent form*. In the *programming background* section, we asked participants for details on their prior programming knowledge, e.g. the time they had been programming, a self-assessment of their skills or the number of programming languages they had already done some work with.

Before the beginning of the study, participants in both studies were randomly assigned to groups. Participants in the first group started with the section that comprised the Athos questions and then went on to the section with the JSprit questions. For Friedberg, this group is referred to as *FbAf (Friedberg, Athos first)*, for Wetzlar the group is referred to as *WzAf, (Wetzlar, Athos first)*.^10^ The other group began with the section containing the JSprit questions and then continued with the section made up of Athos questions. These groups are referred to as *FbJf (Friedberg, JSpirt first)* and *WzJf (Wetzlar, JSprit first)*.^11^

For both the Athos and the JSprit section, participants had exactly 90 minutes to answer all or as many questions as they were able to. After that, the given answers were saved but the survey tool did neither allow for further answers nor modification of any answers given until that point. In the last section, we asked participants for their opinion on several aspects of the study, e.g. which approach they preferred or whether they felt that the learning material for both approaches was of comparable quality.

With the additional questions in the programming background and opinion section, we addressed several threats to validity. A lopsided group assignment which would have posed a threat to the conclusion validity (see Section 6.1), was already addressed by conducting one controlled experiment on each campus and the randomisation of the assignment process. However, it might still have been possible that the majority of highly skilled and passionate programmers was assigned to one group, while the other group was mainly assigned those participants who only possessed little programming skills. The questions on prior programming knowledge allowed us to get an impression whether participants of both groups were equally competent (see Section 5.2).

Even though we spent considerable effort to create high-quality learning material for both approaches (see Section 4.5), inferior training material for either approach might pose an instrumentation threat to the internal validity of the study (see Section 6.2). We therefore asked participants whether they deemed the material for both approaches as being similarly helpful or whether one approach was supported by better material. In the same way, we addressed another instrumentation threat posed by the possibility of unequal time for introduction of the approaches (also see Section 4.5). Though granting the exact same amount of time for the introduction of each approach would have been the most objective and efficient measure to address this threat, it quickly turned out to be impractical since explaining the intricacies of the approaches required a slightly different amount of time for each approach. Moreover, it was important for us to allow participants to ask questions at any given point during the introduction and thus we could not completely control the exact duration of the introduction. For these reasons, we asked participants whether they felt that a similar amount of time was spent for introducing both approaches or whether one approach was granted more time than the other.

### Definition of the Questions and Their Tasks

For the tasks of the survey, it was decided to focus on a problem type natively supported by both approaches. Hence, the tasks of the survey are centred around the VRPTW, a generalisation of the travelling salesman problem (TSP). In addition to the informal description of the VRPTW given in Section 3.2, a formal definition can be found in various works throughout the literature, e.g. in Ombuki et al. (2006) and Baldacci et al. (2008). All questions are publicly available as part of the lab package at github.com/benjaminh20/AthosEvaluation2020.

According to the work presented by Kosar et al. (2010), we also defined three groups of questions, i.e. questions that aimed at testing participants’ ability to *learn*, *perceive* and *evolve* programs of the respective approach. Table 1 provides an overview on the questions we defined for the survey. The first column shows the tag of the question: the first three symbols (QXX) represent the number of the question and the following two letters indicate whether the questions aimed at the network or the agent / vehicle (behavior) part of the respective language. The second column of the table shows the number of (sub) tasks participants had to perform to completely answer the question. The third column informs about the type of the question(s)/tasks. For example, question *Q05NW* was related to the network part of the language and was made up of one multiple-choice task. The fourth column gives a brief description of what participants were expected to do to answer the question. Each question and its respective (sub) tasks were defined for both Athos and JSprit. As an example, question 9 was implemented as Q09ATNW for Athos and as Q09JSNW for JSprit (‘AT’ and ‘JS’ indicating the respective approach).

**Table 1:** Questions and tasks to be solved by study participants (MC = multiple choice, SC = single choice, OT = open text)

Question	Tasks	Type	Description
Learning			
Q01NW	1	MC	*Selection of syntactically incorrect statements:* Participants had to spot syntactical errors in a network specification
Q01AG	1	MC	*Selection of syntactically incorrect statements:* Syntactical errors in an agent behaviour specification had to be spotted
Q02AG	1	MC	*Nonsense statements:* From four given code snippets participants have to select those that do not make sense
Q03ALL	1	SC	*Program with given result:* From three programs one that represented a given tour and network had to be selected
Perceiving			
Q04NW	1	SC	*Sensible result:* Participants had to select a graph that corresponded the network of a given program
Q04AG	1	SC	A tour on a graph that corresponded to a given program had to be selected
Q05NW	1	MC	*Identify language constructs:* Participants had to tick the lines to be changed in a given program to transform the modelled network into a given target network
Q05AG	1	MC	Lines to change the customers for a tour had to be ticked
Q06NW	1	MC	*Programs with equal result:* From four syntactically different programs those that semantically corresponded to a given program had to be selected
Q07ALL	2	SC	*Associate semantic to element:* From four options, the correct purpose of two program elements was to be selected
Q08ALL	3	SC, MC	*Recognise based on comments:* From descriptive comments embedded in a given program, the actual program elements referred to in the comment had to be selected
Evolving			
Q09NW	A3, J4	OT	*Add functionality:* A program with gaps had to be completed according to given specifications
Q09AG	1	OT	Missing customer information had to be added
Q10NW	1	OT	*Remove functionality:* From a given program code was to be removed to achieve a given target state
Q11ALL	2	OT	*Change functionality:* A given program had to be changed according to a graphical specification

The main reason for splitting some questions into several subtasks was that some single-task questions were self-contained whereas others appeared rather incomplete without an additional task. For example, in Q11ATALL, participants were supposed to modify a program in which one single product was distributed to various customers from one single depot. The task was to change the program in a way so that two different products were distributed to the customers from two different depots. Each customer demanded either of the two products (not both). To correctly answer the question (complete the task) participants had to perform two sub-tasks: first, they had to change the network definition (change the demand of customers who now wanted the new product) and introduce the depot. Second, they had to introduce a new agent type for a fleet that was to be stationed at the new depot. The task was thus split into two subtasks. In contrast to that, in Q06ATNW/Q06JSNW participants were presented a network definition defined with the respective approach. Their task then was to pick semantically equivalent network definitions from a set of four programs. This task did not require to be split into subtasks.

**Fig. 6:**
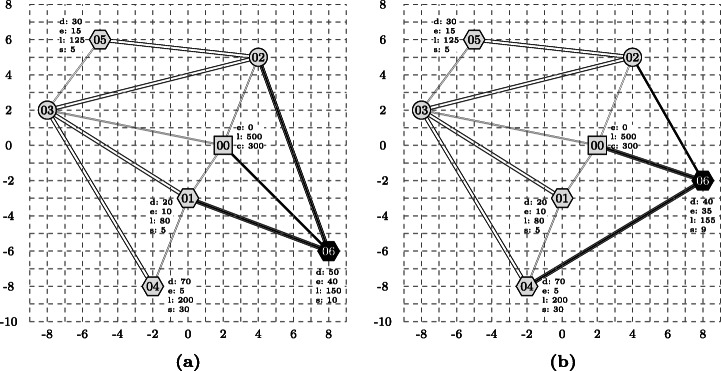
Pictures from the corresponding survey questions Q09ATNW and Q09JSNW: The nodes (depot), (customer), (navigation) and edges — (roads), (highways) represent elements for which pre-defined code was given in the task. Elements emphasized by bold black color had to be added to the program by customers

To allow for a scientifically sound comparison, the questions for both approaches had to be of comparable complexity. In fact, *deviation in the complexity of questions* poses a major threat to validity (see Section 6). Hence, we took utmost care to define the questions so that two corresponding questions had the same structural complexity. An example for this is illustrated in Fig. 6, which is a juxtaposition of the task that was to be performed in the Athos question Q09ATNW (Fig. 6a) and the corresponding JSprit question Q09JSNW (Fig. 6b). In both tasks, participants were presented pre-defined programs that consisted of a number of nodes (which either represented customers with demands, time windows and service times or simple network navigation nodes) and edges (which either represented highways or roads). Elements already present in the respective pre-defined program are depicted in grey colour. The elements printed in bold black colour where elements that participants were supposed to add to the program. As can be seen, the number of pre-defined elements (five nodes, eight edges) and the number of elements to be added (one node, three edges) were the same in both questions so that it is reasonable to assume that both questions were of the same structural complexity. In order to extend the code correctly, participants were supposed to copy marked parts of the code into a text area and add the necessary elements. As can be seen from Table 1, this question is the only one where the number of sub tasks in the two corresponding questions deviated. This is due to the different structure of the two approaches. In order to add the elements, it was sufficient to modify three program parts in Athos, whereas the JSprit code had to be modified at four different places. It is important to note two things here: firstly, this is the only question where we had to have a different number of subtasks; secondly, the higher number of subtasks did not result from a more difficult task that participants had to perform but from the structure of the JSprit code that did not allow to perform the task by only modifying three places of the program. Thus, it remains a valid claim that the questions themselves were of identical structural complexity.

### Point Attribution and Opt-Out Option

In order to be able to statistically analyse the results of the studies, the answers had to be evaluated in order to derive the *score* as a measure for participants’ effectiveness. For this, a scoring scheme had to be developed. We decided for a scheme in which each of the fifteen questions was worth 10 points so that a maximum of 150 points could be achieved. Depending on the structure of the respective question, points were deduced for wrong answers, syntactical and semantic mistakes. We took care to define objective and clear rules that defined how many points for which exact mistake were deduced and we put special effort into defining these rules in a way that no approach was favoured by these rules. This way we sought to avoid biased marking which would have threatened the *reliability of measures* and would thus be a major threat to the validity of this study (see Section 6.1).

The study was conducted throughout the course of regular lectures (see Section 5.2) and it was emphasized both in the preparation and in the informed consent form of the study that participation was completely voluntary. Not only were students allowed to refuse to participate, they were also given the chance to op-out of the questions and tasks of the survey. Generally, the survey system ensured that participants provided an answer for a question (or all of its tasks) before allowing to proceed to the next question. However, each question featured an op-out checkbox that participants could check to move on to the next question without the provision of an answer.

In addition to questions that participants chose to op-out of, questions could also be left unanswered when students exceeded the 90 minute time limit. Finally, questions with text areas in which participants were supposed to enter code using the respective approach could be left blank. It was also possible for participants to enter text that was clearly not intended to solve the respective task, e.g. apologetic statements for not being able to answer the question or complaints on the high difficulty of the task.

### In- and Exclusion Criteria

To ensure that study results were not compromised by participants who did not seriously participate in the study, we applied a set of rules that defined what cases were to be in- and excluded from the study. Firstly, if a data set contained more than seven non-attempts for one of the two approaches, the data set was removed from the results of the study (for example, a data set that contained no non-attempts for Athos but eight non-attempts for JSprit would have been excluded from the study). An answer was deemed as a non-attempt in cases were:

- the opt-out box was checked,
- no answer was given,
- a text area was left empty,
- a text area contained text that was clearly not intended to answer the task.

In a second step, for each group of both studies (i.e. FbAf, FbJf, WzAf, and WzJf), we calculated the difference in the time spent with each approach. For each of the groups we then analysed the resulting distribution by means of a stem and leaf plot and removed outliers. We repeated the same process for every group with the achieved scores. With this approach, we removed those cases in which a participant spent unusually more time with one of the approaches or was unusually more successful with one of the approaches then with the other. Our intention for the removal of these data sets was our conviction that such extraordinary differences in the time spent or the score achieved were most likely not due to differences in the approaches but the result of a loss of motivation or the occurrence of a contingent event not related to the compared languages. These cases would have skewed the within-subjects comparisons presented in Section 5.

In a final step, we only included those cases in which a participant either spent at least 15 minutes or^12^ scored at least 25% of the achievable points with both Athos and JSprit. In other words, we removed all cases in which a participant spent less than 15 minutes without achieving at least 38 points for one or both approaches.

### Transformation of Data

After the evaluation of all obtained datasets and removal of non-attempting participants, some of the data was transformed, i.e. new variables were derived from the existing ones. A simple example is the variable *numberOfLanguages* from the number of ticked checkboxes and the number of additional languages stated in the survey. In Section 5.5, scores and the time required to achieve the respective score will be presented. In order to be able to mathematically prove a connection between the used approach, the achieved score and the time spent answering the questions, a metric that combined both the score and the time spent was required. From the field of behavioural research the *rate correct score (RCS)* (Woltz and Was 2006) was deemed well-suited for the data of the study. The RCS is defined as:

1 $$ RCS = \frac{c}{\sum RT} $$

with *c* denoting the number of correct responses (in a condition) and RT being the reaction time for a trial (Vandierendonck 2017). This definition was applied to the obtained data by interpreting the *c* as the achieved score and $\sum RT$ as the time spent completing the tasks. While the RCS is interpreted as “the number of correct responses per second of activity” (Vandierendonck 2017, p. 654), a simple multiplication by 60 together with the presented interpretation yields the number of points per minute (PPM) spent in answering the questions:

2 $$ PPM = \frac{60 \cdot c}{\sum RT} $$

RCS is equal to another measure (Vandierendonck 2018) known as *throughput* (Thorne 2006). Thorne (2006) notes that throughput is functionally comparable to *number correct per qualified time unit*, a measure they claim to be often applied in tests using pen and paper.

The same measure is also applied by Kosar et al. (2012, 2018) where it is referred to as participants’ *efficiency*. This is because a high value for either RCS, PPM and throughput can be regarded as an indicator for a participant working efficiently with the respective approach: A participant who achieved 120 points in 45 minutes (RCS: .044, PPM: 2.667) certainly solved the tasks more efficiently than a participant who spent the full 90 minutes on the tasks and also scored 120 points (RCS: .022, PPM: 1.333). However, it is debatable whether a result of 20 points scored in 4 minutes (RCS: .083, PPM: 5), though more efficient, is more desirable than the aforementioned 120 points scored in 45 minutes. For this reason, the presentation of the study results in Section 5 gives insight on the scores alone and in relation to the time spent solving the tasks.

### Statistical Comparison of the Approaches

**Fig. 7:**
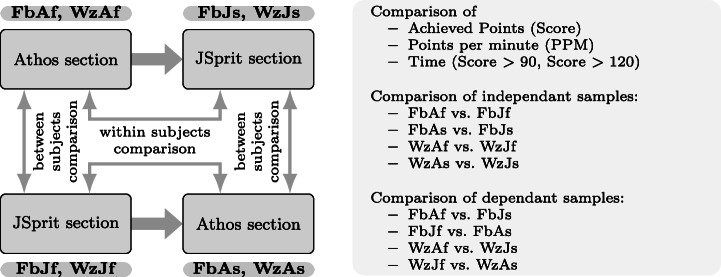
Different comparisons of the obtained results: Results obtained from two independent samples (Athos first vs JSprit first and Athos second vs. JSprit second) as well as two dependant samples (Athos first vs JSprit second and JSprit first vs Athos second) were compared

The design of our study allows for two directions in which the results of both approaches can be compared. As is illustrated in Fig. 7, it is possible to compare *dependent* results *within* the respective groups (within-subjects comparison), i.e. calculate the delta of points achieved with the first and the second approach. This can be done on the basis of each single data set as well as in an aggregated way, e.g. by calculating the mean score for the first and the second approach and then the difference of the two. The difference can then be tested for statistical significance by application of the Wilcoxon signed-ranks test.

The other direction in which the results for two approaches can be compared is to compare *independent* results *across* the two groups of one study (between subjects comparison). Here the results of both approaches used as a first approach (and also as a second approach) are compared. Statements on a statistically significant difference in the results can be made by application of the Mann-Whitney U test.

Figure 7 also depicts the study results that were compared: Not only did we compare the results in terms of the score achieved by participants, but also in terms of points in relation to time by testing the achieved PPM.

## Results

### Application of Exclusion Criteria

In Section 4.9, we presented the in- and exclusion criteria of the study. Table 2 summarises the results of the application of these criteria to all submitted cases. The first column shows the group to which the respective filter/measure was applied and the second column presents the original number of cases for each group.

**Table 2:** Number of cases excluded and included in the study

Group	ORG	Non-attempt filter I				Deviation		II	INC
		NA_At_	NA_Js_	OR*	AND	TME	SCR	MEF	
FbAf	63	16	23	24	15	4	0	0	35
FbJf	55	16	14	17	13	0	0	4	34
WzAf	19	1	2	2	1	0	2	1	14
WzJf	22	1	0	1	0	1	2	0	18

For each group the table shows: the original cases (ORG); the number of cases with more than seven unanswered questions for Athos (NA_*At*_), JSprit (NA_*Js*_), either approach (OR*), and both approaches (AND); the number of cases with skewed times spent (TME), skewed scores (SCR), the number of cases without minimal effort (MEF), and the number of included cases (INC)

Columns three to six show the number of participants who opted out of more than seven questions for Athos, for JSprit, for either of the two, and for both. As was explained, a case was excluded if a participants opted out of more than seven questions for either approach so that the fifth column is the one that gives the actual number of cases excluded due to the number of unanswered questions. The sixth column is included in the table to show that most cases that were excluded from the study exceeded the number of seven non-attempts for both approaches.

The seventh and eighth column show the number of cases that were excluded due to an unusually high deviation (in relation to the observed deviation of the sample population) between the times spent and the scores achieved with the approaches, respectively. The ninth column shows the number of cases that were removed because participants spent less than 15 minutes on one (or both) of the approaches without achieving at least 38 points. Finally, the last column shows the number of included cases for the respective group.

Overall, the data in the table show two important facts: firstly, the limit to allowed non-attempts was the one on which most exclusions were based. This means, that the majority of participants either just skimmed through the questions without answering them, or they put in the required effort for both approaches. Secondly, it can also be clearly seen that in the Friedberg study there were substantially more cases excluded than in the Wetzlar study course. Given that the only compensation was that participation was announced to be a perfect training towards the final exam of the respective module, this was to be expected. Students in Friedberg have more freedom in defining their individual curriculum than students enrolled in the co-operative study-program offered in Wetzlar. Thus, only a certain proportion of students attending a module actually sit the final exam. This fact is reflected in the data presented in the table and we do not consider the observed number of drop-outs a threat to the validity of our study.

### Prior Programming Knowledge

**Fig. 8:**
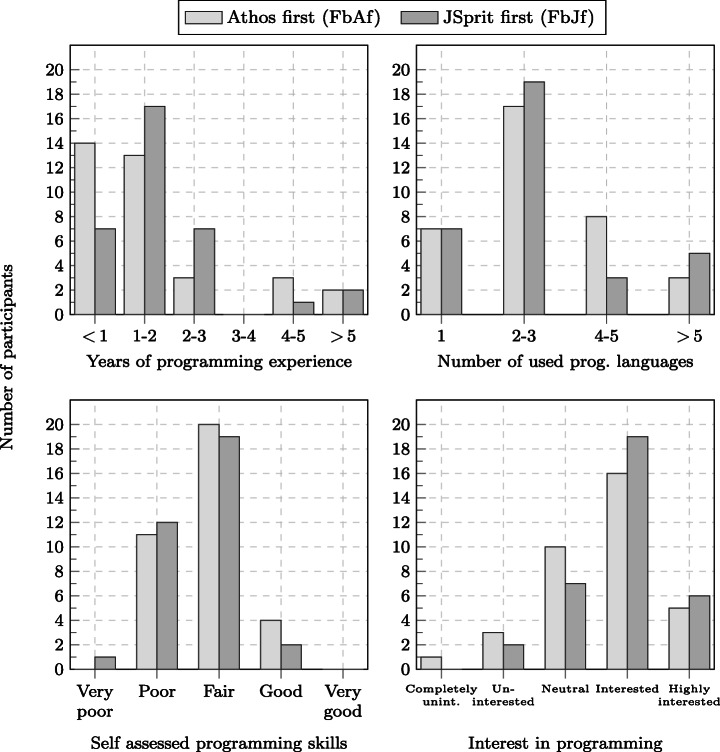
Comparison of participants’ prior programming experience, number of programming languages used, self-assessed programming skills and general programming interest between the Friedberg group using Athos first () and the Friedberg group using JSprit first ()

**Fig. 9:**
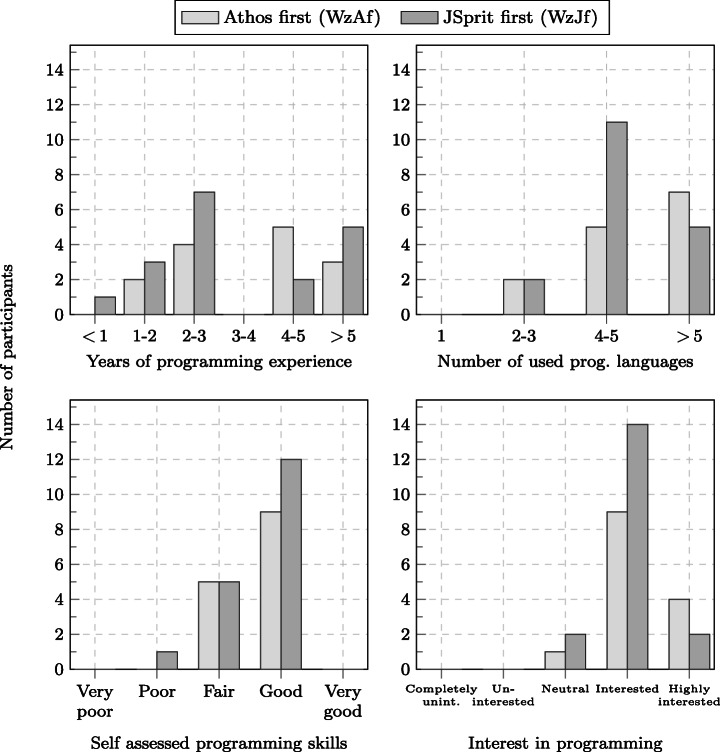
Comparison of participants’ prior programming experience, number of programming languages used, self-assessed programming skills and general programming interest between the Wetzlar study group using Athos first () and the Friedberg study group using JSprit first ()

In order to be able to put the language evaluation results into context, we presented participants with questions on their programming background. Figure 8 compares the answers given by the two groups of the Friedberg participants, Fig. 9 does the same for the two groups from the Wetzlar study. Overall, the results show that a) participants from the respective groups of both studies (Athos first and JSprit first) possessed comparable prior programming skills and knowledge, and b) participants from Friedberg had less programming experience than those from Wetzlar.

In terms of years of programming experience, Fig. 8 shows that in Friedberg the JSprit first group (FbJf) was slightly more experienced than the Athos first group (FbAf). Only seven members of the JSprit first group (≈ 21% of all group members) stated to have less than a year of programming experience while twice as many members of the Athos first group (40%) claimed to have less than 12 months of programming experience. However, in both groups the vast majority of participants did not have more than two years of programming experience (more than 70% in both groups). Programmers with more than two years of experience were rare in both groups: only eight members of the Athos first group and ten members of the JSprit-first group stated to have more than two years of experience in the usage of computer languages. Based on these data, it can be concluded that most participants in the study conducted in Friedberg had only just started their programming careers.

Regarding the other questions, results for both groups were generally very similar. In both groups, most members had experience with two or three computer languages and there were exactly seven participants in each group who had only worked with one language. The two groups also had a similar number of participants that had used four or even more languages, though the Athos first group had slightly more participants in that category. Asked for a brief self assessment, in both groups the majority stated to consider themselves fair programmers. In both groups there was a strikingly high number of participants who expressed doubt in their own programming skills: As many as 13 participants from the FbAf and 16 from the FbJf group thought of themselves as poor or even very poor programmers. The majority of both groups was either interested or highly interested. While both groups also had a surprisingly high number of participants with only moderate interest, only a negligible number of participants stated their disinterest in the subject.

Figure 9 illustrates the results of the groups from the Wetzlar study. As regards the years of experience, neither group appears to have had a definitive advantage over the other. Although the JSprit first group consisted of more participants who had been programmers for more than five years, the Athos first group on the other hand had more members with four to five years of experience. While there were a few more members with 2–3 years of experience in group WzJf, it is also important to consider that the group consisted of four more members than the WzAf group.

Looking at the answers to the other questions, the results for both groups are also rather similar. The distinct difference in the number of group members who had used 4–5 programming languages can be assumed to result from the different group sizes. In any case, both groups nearly completely consisted of members who had experience with at least four languages. Only a minority of two participants in each group stated to have used only 2–3 different programming languages.

Members of both groups expressed similar confidence in their programming skills. The majority of both groups stated to be good programmers. However, in both groups there were five participants who only considered themselves to be fair programmers. In both groups, nearly all participants were either interested or even highly interest in the subject of programming.

A comparison of the groups from Friedberg and Wetzlar clearly shows that participants from Wetzlar were more experienced, active and confident in the usage of programming languages. The mode and median value for three of the prior knowledge questions are reported in Table 3. Overall, for each of the three questions, both the mode and mean value of the combined Wetzlar study group were one level above the mode and mean of the combined Friedberg study group. Not reported in the table are the mode and median of the question on participants’ interest in programming. For both the Friedberg and the Wetzlar study, the most frequent answer in both groups was that participants were “interested” in the subject of programming, which was also the median value.

**Table 3:** Comparison of study groups

	Experience		Languages		Skills	
	Mode	*Mdn*	Mode	*Mdn*	Mode	*Mdn*
Friedberg^a^						
Overall	1 – 2	1 – 2	2	3	Fair	Fair
Athos first	< 1	1 – 2	2	2	Fair	Fair
JSprit first	1 – 2	1 – 2	3	3	Fair	Fair
Wetzlar^b^						
Overall	2 – 3	2 – 3	4	5	Good	Good
Athos first	4 – 5	4 – 5	6	5.5	Good	Good
JSprit first	2 – 3	2 – 3	4	4.5	Good	Good

^a^ *N* = 69, *n*_FbAf_ = 35, *n*_FbJf_ = 34

^b^ *N* = 32, *n*_WzAf_ = 14, *n*_WzJf_ = 18

These results allow two important conclusions: firstly, the validity of a *between-subjects* comparison, i.e. a comparison between the results of two groups of the same study, is not threatened by a *lopsided group assignment* (see Section 6) since members of the two respective groups in both studies had comparable programming knowledge. Secondly, the different knowledge levels observed for participants of the two studies from Friedberg and Wetzlar allow the assumption that participants from Friedberg can be considered to represent domain experts with only limited programming experience, whereas participants from Wetzlar are more suitable to represent junior software developers who possess a well-grounded background in programming. This provides some more context for the results presented in the following sections.

### Score Distribution

As was mentioned in Section 4.2, we intended to find out whether Athos has the potential to facilitate comprehension of VRP models. For this reason, we formulated the hypothesis that the application of Athos had no effect on the test results (scores) achieved by study participants. In this section, we report the scores achieved by the groups of the two conducted studies. In addition, we will present the outcome of various statistical tests performed on the obtained data and discuss their implications on our hypothesis.

**Fig. 10:**
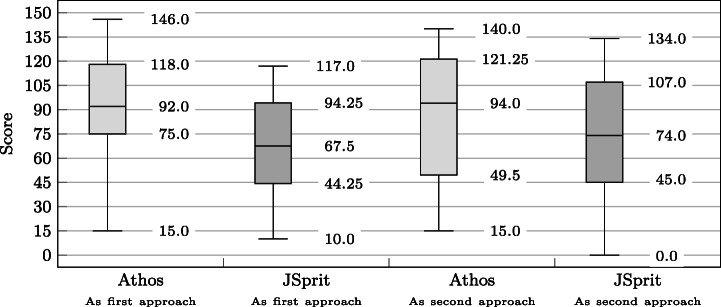
Boxplot showing the distribution of the scores achieved by participants of the Friedberg study (*n*_FbAf_ = 35, *n*_FbJf_ = 34)

Figure 10 presents an overview of the results obtained in the Friedberg study. What is particularly noticeable is the marked difference in the scores for both languages used as a first approach: the median value for Athos as a first approach surpassed the median of JSprit by more than 24 points. Even the lower quartile for Athos exceeds the median of JSprit by 7.5 points. It thus appears that for users with little programming experience, Athos had a substantially positive effect on the observed scores. A comparison of the results for both languages used as a second approach also shows that on average users scored higher when using Athos. However, the differences in the achieved scores do not appear to be as compelling as those observed when both languages were used as first approaches.

The group that started with Athos exhibited declining scores when JSprit was used as a second approach. The median, for example, dropped from 92 points to a mere 74 points. Even the lower quartile with Athos first is above the median for JSprit as a second approach. The top result with Athos first was a remarkable 146 points – with JSprit second the best result dropped by 12 points to a score of 136. Conversely, when participants started with JSprit and used Athos as a second approach, there was a remarkable increase in points. The median drastically rose from 67.5 points to 94 points and the upper quartile increased from 94.25 points to an impressive 121.25 points. With Athos second the top result was considerably higher than the best result achieved with JSprit first: with JSprit, the best participant scored 117 points which was 23 points less than the best score achieved with Athos second.

**Fig. 11:**
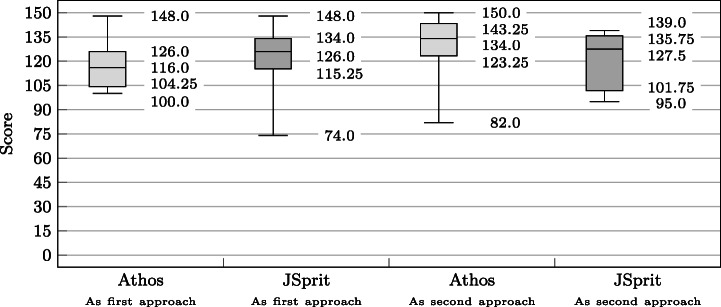
Boxplot showing the distribution of the scores achieved by participants of the Wetzlar study (*n*_WzAf_ = 14, *n*_WzJf_ = 18))

Figure 11 gives an overview on the scores achieved in the Wetzlar study. What is immediately obvious is the fact that participants in this study achieved higher scores than participants from the Friedberg study. This was to be expected due to the fact that participants from Wetzlar had distinctly more experience in the field of programming (see Section 5.2). Looking at the respective interquartile ranges, it is also apparent that the results were less dispersed than those in the Friedberg study. While there were exceptionally high scoring and exceptionally low scoring participants in both groups, the majority of participants scored in the range of 100–140 points.

Juxtaposing the results for both languages as the first approach, the results for JSprit were somewhat better than those for Athos. With both approaches the best score was 148 points and with Athos no participant scored less than 100 points which is 26 points above the lowest score achieved with JSprit. However, the median score with JSprit is 10 points above the median score achieved with Athos and both the upper and lower quartile comparison are in favour of JSprit.

On the other hand, if the languages were used as the second approach, the results for Athos were more favourable than those observed for JSprit. With Athos as the second approach, one participant even scored a perfect 150 points whereas the highest score with JSprit as the second approach was 139 points. The median value with Athos as the second approach was slightly above the median value for JSprit.

A within-subjects comparison of both approaches shows that participants who started with Athos were able to moderately improve their results with JSprit as a second approach. The median score in that group rose by 11.5 points from 116 to 127.5 points. However, the top result dropped from 148.0 to 139.0 points and the lower quartile also dropped by 2.5 points. On the other hand, a similar improvement with the second approach could also be observed when Athos was used second. Here, the median improved by 8 points and the other quartiles as well as both whiskers are also higher than those observed for JSprit as the first approach.

### Statistical Significance Tests of the Scores

For the *within-subjects comparison*, we used the Wilcoxon signed-rank test. Table 4 reports on the results obtained. In the Friedberg study, both groups achieved significantly better scores with Athos than with JSprit. The p-values of the tests for both groups are well below the 5% significance level. In Wetzlar, the group that started with Athos performed better when using JSprit, however, the result was not statistically significant. By contrast, the group that started with JSprit performed significantly better with Athos as the second approach with a p-value of only 0.007 and thus high statistical certainty.

**Table 4:** Within-subjects comparison of the achieved score using the Wilcoxon signed-rank test

	*n*	Ties	*Mdn*_Score_		*W*	*Z*	*p*
			Athos	JSprit			
Fb Athos first	35	0	92.0	74.0	84.5	− 3.776	0.000*
Fb JSpirt first	34	0	94.0	67.5	84.0	− 3.651	0.000*
Wz Athos first	14	0	116.0	127.5	38.0	− 0.912	0.362
Wz JSprit first	18	1	134.0	126.0	19.5	− 2.701	0.007*

The significance threshold (alpha) was defined to be 0.05

To statistically test the results of the *between-subjects score comparison*, we used the Mann-Whitney U test for which the results are displayed in Table 5. In Friedberg, Athos as a first approach resulted in significantly higher scores than JSprit as a first approach. As a second approach, Athos was also the approach for which – on average – higher scores were observed, though not at a statistically significant level. In Wetzlar, notwithstanding whether they were used as a first or second approach, neither the use of Athos nor the use of JSprit resulted in significantly superior scores.

**Table 5:** Between subjects comparison of the achieved score using the Mann-Whitney U test

	*N*		*Mdn*_Score_		*Mdn*_Rnk_		*U*	*Z*	*p*
	Athos	JSprit	Athos	JSprit	Athos	JSprit			
Fb as first	35	34	92.0	67.5	42.30	27.49	339.5	− 3.067	0.002*
Fb as second	34	35	94.0	74.0	37.97	32.11	494.0	− 1.212	0.225
Wz as first	14	18	116.00	126.0	14.25	18.25	94.5	− 1.200	0.230
Wz as second	18	14	134.00	127.5	18.22	14.29	95.0	− 1.179	0.251

The significance threshold (alpha) was defined to be 0.05

These results show that Athos has the potential to facilitate users’ effectiveness in program comprehension. Three out of four within-subjects comparisons show statistically significant improvements in the achieved scores for the Athos approach (FbAf, FbJf, WzJf). Only one comparison was moderately in favour of JSprit (WzAf), i.e. not at a statistically significant level. Of the between-subject comparisons, the only one with a statistically significant result (Friedberg, first approach) clearly favoured Athos as the more effective approach. Two more comparisons also were in favour of Athos (Friedberg, second approach; Wetzlar, second approach), though not at a statistically significant level. Only one comparison (Wetzlar, first approach) was in favour of Athos but also not at a level of statistical significance.

In three out of four tests for the Friedberg study group, the application of Athos elicited a significant increase of achieved scores. Though there is no definite explanation as to why the positive effects on users’ effectiveness somewhat diminished when both Athos and JSprit were compared as second approaches, a reasonable assumption is that learning and exhaustion effects may have had a strong influence on the outcome. This is especially plausible given that participation in the study was completely voluntary and there was no special incentive for participants to keep the focus on the highest level for the entire study duration of up to three hours.

The results of the Wetzlar study group show a significant increase in scores when JSprit was the first and Athos the second approach. This provides reliable evidence for the claim that Athos has the potential to improve the effectiveness of more experienced programmers in comparison to traditional approaches. When both languages were used as a second approach by the more experienced programmers from Wetzlar, the observed scores with Athos were also moderately better than those achieved with JSprit, but the improvement was not enough to be statistically significant.

In case both languages were used as a first approach, the results for Athos were to some extent inferior compared to those achieved with JSprit. The same holds true for a within-subjects comparison that favours JSprit as the second approach over Athos as the first approach. These results show that despite its general potential to enhance users’ benefits, there are certain situations in which more experienced users might not benefit from the usage of Athos. But here it is important to note that it was to be expected that the positive effects of Athos might be less pronounced among a group of more experienced programmers. As Barišić et al. (2014) noted, the positive effects of a DSL application are generally less distinct among participants with sophisticated programming knowledge. Two additional factors that might have contributed to these results are the fact that participants in the Wetzlar study had to learn and apply Athos at the very same day (see Section 4.5) which might have been too short of a time to catch up with mostly 2–3 years of GPL experience. Secondly, it is possible that the presented questions were not complex enough for this group, since we used the same questions for the comparably inexperienced participants from Friedberg. As is reported by Kosar et al. (2018), improvement of results related to the application of a DSL are more distinguishable for tasks of high complexity.

### Overview on Results Related to Participants’ Efficiency

The second research question asked whether Athos enhanced modellers’ efficiency in model comprehension and creation. In Section 4.10, we defined the achieved PPM as a measure for model comprehension efficiency. The next sections will present the observed efficiency results of both studies.

#### Overview on Efficiency Results from Friedberg

**Fig. 12:**
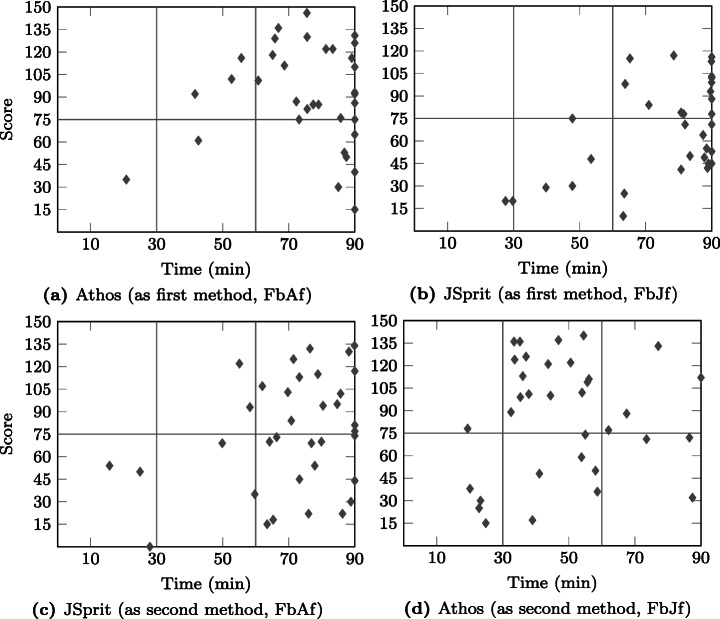
Scatterplots of the scores achieved in relation to required time for Athos and JSprit used as a first and second method (data from the Friedberg study with less experienced programmers)

Figure 12 illustrates the amount of time participants from the two groups of the Friedberg study spent at the given language section as well as the final score they achieved. Each of the four plots is divided into six parts. The part on the bottom left, for example, contains data points representing those participants, that spent between 0 and 30 minutes trying to solve the tasks with the respective approach and achieved less than 75 points. Data points in the upper right part represent those participants who scored more than 75 points within a time frame of 60 to 90 minutes. Hence, the ideal approach would have the majority of data points located in the upper left section, i.e. participants would have been awarded more than 75 points in less than 30 minutes.

#### Within-Subjects Comparison

In the group that started with Athos as a first approach, efficiency dropped to some extent when JSprit was used as a second approach: with Athos, 24 participants scored above 75 points within 60 to 90 minutes. With JSprit, the number of participants who scored over half the points in 60 to 90 minutes reduced to only 15. However, with JSprit as their second approach, there were six participants who missed the 75 points mark by only six or less points.

Conversely, the group that started with JSprit as their first approach exhibited a massive efficiency gain with Athos as the second approach: with JSprit, only one single participant recorded 75 points in less than 60 minutes. In stark contrast, with Athos as the second approach, as many as 17 participants scored 75 points or above in less than 60 minutes.

#### Between Subjects Comparison

In order to compare how participants performed using either Athos or JSprit as a first approach, the two upper plots have to be analysed. A contrasting juxtaposition of the two plots suggests that participants scored higher in less time when using Athos as the first approach: with Athos, as many as 27 of the 35 participants (i.e. upwards of 75% of all participants from that group) scored more than 75 points. Three participants who started with Athos managed to do so in a time window of 30 to 60 minutes. With JSprit first, only 15 of all 34 participants (a mere 44.1% of that group) achieved more than half of all points and only one single participant achieved the score in a 30 to 60 minutes time window. Looking at the numbers of those who did not score at least half of all available points, with Athos only 8 (22.9%) participants fell short of achieving 75 points. With JSprit, on the other hand, as many as 19 participants (55.9%) lost more than half of all points. With Athos as the first method only six participants (17.1%) spent more than an hour on the tasks without achieving at least 75 points. By contrast, with JSprit first 14 participants (41.2%) spent over 60 minutes at the tasks and achieved less than 75 points. Used as a first approach, neither Athos nor JSprit saw a participant scoring close to 75 points in less than 30 minutes.

A comparison of the results for both approaches used as a second approach also leads to the conclusion that participants of the Friedberg study were more efficient when using Athos: with JSprit, only 17 out of 35 participants scored higher than 75 points (48.6%); with Athos, 21 out of 34 participants achieved more than 75 points (61.8%). From these participants, merely two JSprit users achieved their score within 60 minutes compared to 17 Athos users who achieved more than half of the points within a time frame of up to 60 minutes. With Athos as the second approach, there was even one participant who scored more than 75 points in less than 30 minutes (78 points in 19 minutes and 21 seconds). With JSprit second, the closest a participant came to achieving 75 points in under 30 minutes was a participant who scored 54 points in 15 minutes and 44 seconds.

#### Efficiency Results from Wetzlar

**Fig. 13:**
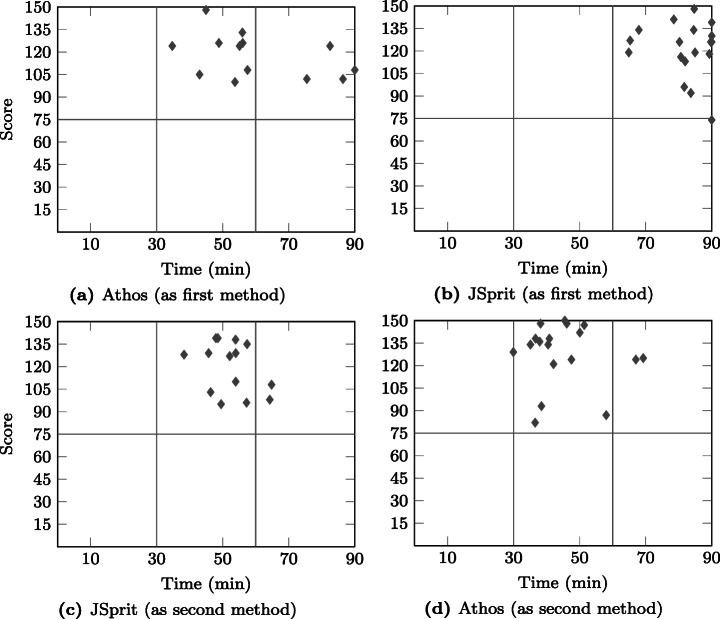
Scatterplots of the scores achieved in relation to required time for Athos and JSprit used as a first and second method (data from the Wetzlar study with more experienced programmers)

Having compared the efficiency results for Athos and JSprit when used by participants with comparably little programming experience, it is also interesting to see the results for both approaches applied by more experienced participants. Figure 13 illustrates the score and survey time of participants who took part in the Wetzlar study.

#### Within-Subjects Comparison

The two diagrams on the left-hand side of Fig. 13 illustrate that the group which started with Athos was noticeably more efficient with JSprit as their second approach. While the recorded scores seemed somewhat similar, the required time to achieve these scores was markedly reduced with JSprit. With Athos, all 14 participants scored more than 75 points, but only 9 (64.3%) did so in less than an hour. With JSprit, still all 14 participants scored more than half of the achievable points; with JSprit as the second approach, however, 12 participants (85.7%) finished in under 60 minutes.

Considering the right-hand side of Fig. 13, it is obvious that the group that used JSprit first and Athos second also exhibited a substantial increase of efficiency from the first to the second approach. Here, the increase was even more distinct: with JSprit all but one participant, i.e. 17 out of 18 (94.4%), scored above 75 points but not a single participant managed to do so in under 60 minutes. With Athos as the second approach, all 18 participants passed the 75 points mark and as many as 16 of these participants did so in under an hour. One participant even recorded 127 points in less than 30 minutes.

#### Between-Subjects Comparison

The plots in the upper half of Fig. 13 clearly show that a considerable number of participants solved the Athos tasks in less time than the respective JSprit tasks and still achieved a score of 75 or higher: With Athos, all 14 participants were awarded more than 75 points and 9 of these participants (64.3% of the entire sample) even managed to achieve their respective score in 30 to 60 minutes. With JSprit, 17 of 18 participants (94.4%) passed the 75 points mark but none of these participants finished in less than 60 minutes.

When used as a second approach, the time participants spent solving the tasks with the respective approach appear to be rather similar: With Athos second, 16 out of 18 participants (88.9%) scored more than 75 points in less than 60 minutes; with JSprit, 12 out of 14 participants (85.7%) collected more than 75 points in under 60 minutes. Though the survey times for the two approaches used as a second approach are not as far apart as when used as a first approach, participants who used Athos still spent less time than those who used JSprit. One of the participants who used Athos second even achieved more than 75 points in less than 30 minutes (127 points in 29 minutes and 56 seconds). With JSprit second, the closest a participant with over 75 points came to the half-hour mark was a participant who scored 128 points in 38 minutes and 18 seconds. Looking at the scores, it seems that Athos caught up with JSprit, especially in the number of high-scoring participants: With Athos second, 8 participants (44.4%) scored 135 or more points; With JSprit only 4 participants (28.6%) accomplished the same feat.

### Test Results for Participants’ Efficiency

The results of the conducted significance tests confirm the observations made in the previous section. Table 6 summarises the results of the *within-subjects significance tests* for which we applied the Wilcoxon signed-rank test. In Friedberg, there was a significant reduction in achieved PPM when participants used Athos first and JSprit second. The other Friedberg group that had JSprit as the first approach displayed a significant increase in achieved PPM with Athos. In the Wetzlar study, there was a significant growth for either language when used as a second approach. There is however, a difference in the observed p-values for both within-subjects comparisons: the growth observed with JSprit as the second approach barely remains below the 0.05 threshold for statistical significance; in the other group where Athos was used as the second approach, the observed p-value of 0.000 leaves no doubt that Athos as a second approach substantially enhanced users efficiency in model comprehension and creation.

**Table 6:** Within-subjects comparison of the achieved PPM using the Wilcoxon signed-rank test

	*n*	Ties	*Mdn*_PPM_		*W*	*Z*	*p*
			Athos	JSprit			
Fb Athos first	35	0	1.2222	1.1218	152.0	− 2.670	0.008*
Fb JSpirt first	34	0	1.8913	0.8277	17.0	− 4.796	0.000*
Wz Athos first	14	0	2.0625	2.3719	21.0	− 1.977	0.048*
Wz JSprit first	18	0	3.0417	1.4412	0.0	− 3.724	0.000*

The significance threshold (alpha) was defined to be 0.05

The results of the *between-subjects significance tests* are provided in Table 7. In the Friedberg study, both groups were significantly more efficient when using Athos: with our DSL – both as a first and as a second approach – the achieved PPM proved to be significantly higher than those obtained with JSprit.

**Table 7:** Between subjects comparison of the achieved PPM using the Mann-Whitney U test

	*N*		*Mdn*_PPM_		*Mdn*_Rnk_		*U*	*Z*	*p*
	Athos	JSprit	Athos	JSprit	Athos	JSprit			
Fb as first	35	34	1.2222	0.8277	42.77	27.00	323.0	− 3.265	0.001*
Fb as second	34	35	1.8913	1.1218	42.79	27.43	330.0	− 3.181	0.001*
Wz as first	14	18	2.0625	1.4412	20.43	13.44	71.0	− 2.089	0.037*
Wz as second	18	14	3.0417	2.3719	19.89	12.14	65.0	− 2.317	0.020*

The significance threshold (alpha) was defined to be 0.05

In the Wetzlar study, results also hint at an increased efficiency of participants’ program comprehension and creation when using Athos. While the previous section showed, that if only the achieved scores were considered, the results of the group that used JSprit first were moderately superior to those who used Athos as a first approach. Putting these scores in relation to the time required to achieve them, however, shows that Athos was the significantly more efficient approach. Comparing both languages when applied as a second approach, also shows significantly superior efficiency results for Athos.

The presented results of participants’ efficiency (PPM) show that Athos substantially enhances the efficiency of end users. This is especially true for end users with limited knowledge in software development as the results from the Friedberg study group suggest. In all four conducted tests participants exhibited significantly increased efficiency results when using Athos. This clearly supports the hypothesis that Athos can positively affect programmers’ efficiency in model comprehension and creation.

The results of the Wetzlar study provide evidence in support of the claim that Athos has the potential to increase the efficiency of programmers who are experienced in the usage of GPLs and application libraries. Even though the group that started out with Athos and then used JSprit as a second approach exhibited significantly improved results with JSprit second, the three remaining tests were clearly in favour of Athos as the more efficient approach.

The result from the *within-subjects* comparison suggest that learning effects seem to be an important factor in the achieved efficiency. In both groups the second approach yielded superior results in terms of PPM. Performing a *between-subjects comparison*, however, shows that Athos significantly increases participants efficiency.

### Results on Observed User Satisfaction

End user and/or domain-expert appreciation is a crucial factor that determines the success or failure of any DSL (Kahlaoui et al. 2008). Hence, we wanted to gain some insight on how participants perceived the usage of both languages. In order to be able to answer the question of whether participants felt more satisfied with Athos than with a traditional approach for modelling VRPTWs, we designed the final part of the survey so that they could express their opinion on both languages. More precisely, for both languages participants were presented five statements and asked to express their degree of agreement to the respective statement using a five-point Likert scale.

The answers obtained from both studies leave no doubt that participants preferred working with Athos over using JSprit to model/program VRPTWs. In both studies, participants expressed a similarly high appreciation for our DSL. By comparison, participants expressed distinctly less satisfaction with the JSprit approach. As was to be expected, the participants from Friedberg with less Java experience were even more skeptical towards JSprit than participants from the Wetzlar study.

**Fig. 14:**
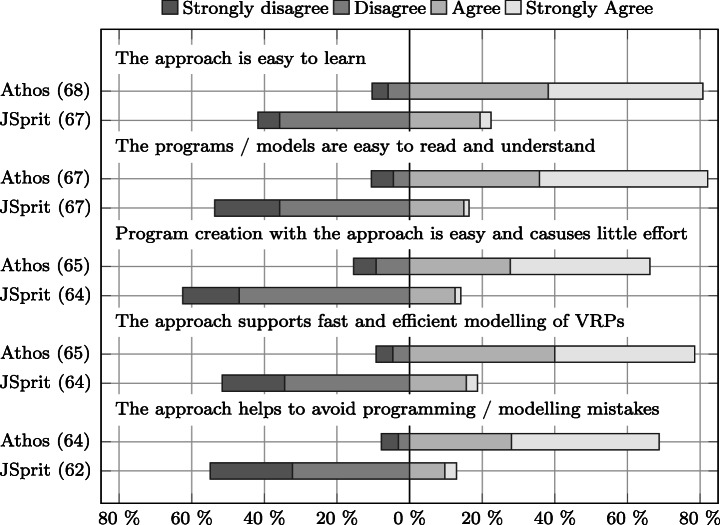
Overview how participants from Frieberg perceived working with Athos and JSprit

Figure 14 illustrates the results obtained from the Friedberg study group. From 68 participants who answered the question whether they agreed that Athos was easy to learn, 81% gave their consent to this statement. For JSprit the same question was answered by 67 participants but only 22% felt that JSprit was easy to learn. A considerable 82% stated that Athos models were easy to read and understand whereas only 16% stated the same for JSprit. Nearly two out of three participants would affirm that modelling with Athos caused them little effort. With JSprit, however, not even one out of five participants was under the impression that model creation with JSprit was easy. A similarly high percentage was positive towards Athos’ support of fast and efficient modelling (79%) as well as its potential in helping to avoid mistakes (69%). With JSprit participants appeared distinctively more skeptical to support these claims with only around 19% attesting to a potential for efficient modelling and an even lower percentage (13%) feeling that the usage of JSprit could help to avoid mistakes.

**Fig. 15:**
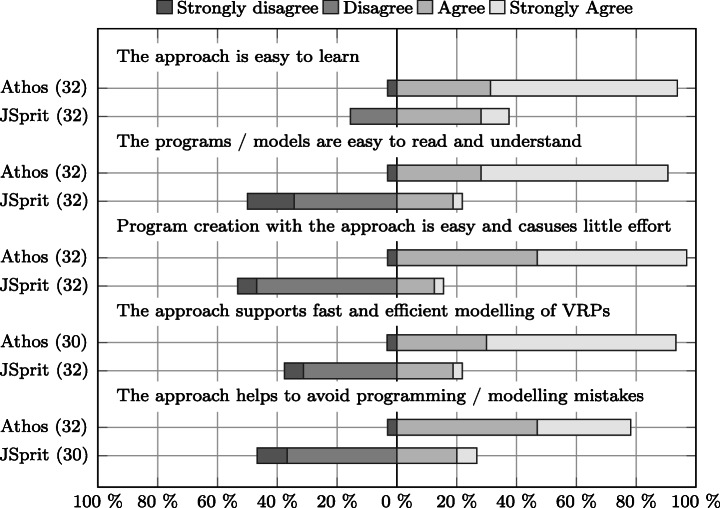
Overview how participants from Wetzlar perceived working with Athos and JSprit

The results from the study group in Wetzlar are illustrated in Fig. 15. As can be seen, the results obtained in this study are similarly positive towards Athos with agreement percentages ranging from 78% (avoidance of mistakes) to 97% (easy program/model creation). Compared to participants from Friedberg, participants from the Wetzlar study appeared to be slightly more positive towards JSprit, though agreement percentages ranging from 16% (easy program/model creation) to 38% (easy to learn) remained low in comparison to the results obtained for Athos.

### Statistical Tests on Observed User Satisfaction

To test the obtained results for statistical significance, we applied the Wilcoxon signed-rank test on the answers for each statement. The test results are summarised in Table 8. The test results unequivocally prove that users were more satisfied with Athos than they were with JSprit. For both the Friedberg and the Wetzlar study, Athos received a significantly higher level of agreement for every single positive statement on a given language characteristic.

**Table 8:** Summary of the Wilcoxon signed-rank test applied to the obtained agreement levels to statements on several language characteristics

	*n*	Ties	*Mdn*_Likert_		*W*	*Z*	*p*
			Athos	JSprit			
Friedberg							
Easy to learn	67	11	4.0	3.0	145.0	− 5.416	0.000*
Easy to read	66	10	4.0	2.0	109.0	− 5.686	0.000*
Easy to create	64	12	4.0	2.0	132.0	− 5.125	0.000*
Fast and efficient	63	12	4.0	2.0	82.0	− 5.505	0.000*
Avoidance of mistakes	62	13	4.0	2.0	61.0	− 5.537	0.000*
Wetzlar							
Easy to learn	32	4	5.0	3.0	28.5	− 4.071	0.000*
Easy to read	32	1	5.0	2.5	34.0	− 4.234	0.000*
Easy to create	32	2	4.5	2.0	26.0	− 4.343	0.000*
Fast and efficient	30	1	5.0	3.0	25.5	− 4.233	0.000*
Avoidance of mistakes	30	5	4.0	3.0	43.0	− 3.254	0.000*

Levels of agreement were expressed with a five-point Likert scale that ranged from 1 (Strong disagreement) to 5 (strong agreement)

The significance threshold (alpha) was defined to be 0.05

These results provide substantial evidence that Athos increases satisfaction levels of both language users with little programming experience as well as more experienced users at a junior developer level. Both type of users recognise and appreciate the effects that Athos has on the comprehension and creation processes required to understand and write models for VRPTWs.

Not only did participants from both studies express their perception that Athos was easier to be learned, they also left no doubt that they felt that Athos models were easier to be read and understood. What is more, participants did not only prefer Athos in order to read and understand already existing models, they also clearly expressed their preference for Athos when it comes to active creation of VRPTW models: participants stated that they considered model creation with Athos comparatively easy and effortless as well as fast and efficient. Finally, participants of both studies also stated their trust in the concise language syntax in supporting them in the creation of correct models.

As was already mentioned, a high level of user acceptance is a necessary requirement for the success of any DSL. Even though the presented levels of agreement are subjective perceptions of several language aspects rather than objective measurements of the respective language characteristics, they are no less important. If users experience the usage of a language in a negative way, it is likely that this will affect the results they produce with the repudiated language. On the other hand, if users feel that a given language is a helpful tool for a certain task, it is likely to positively affect the produced outcomes. This might even be reinforced by an easier and faster learning process that is not hindered by an inner distaste.

### Quality of Teaching

In order to address deviations in the quality of the training material and the time granted for learning the approaches as possible threats to the internal validity of the study, we took care to not bias the results through either of the two means (see Section 6.2). To further ensure that our efforts were successful, we wanted to gain insight on whether they were recognised by participants of the two controlled experiments. To this end, we asked participants whether they considered the learning material provided for both approaches to be of similar quality and whether they had the impression that they were granted a comparable amount of time to learn both approaches. The results are presented in Table 9.

**Table 9:** Summary of the Wilcoxon signed-rank test applied to the obtained agreement levels to statements on several language characteristics

Question	Athos		Identical		JSprit		No answer	
	Frq	%	Frq	%	Frq	%	Frq	%
Friedberg								
Better learning material	4	5.8	60	87.0	0	0.0	5	7.2
More time to learn	2	2.9	58	84.1	6	8.7	3	4.3
Wetzlar								
Better learning material	1	3.1	31	96.9	0	0.0	0	0.0
Easy to read	0	0.0	30	93.8	2	6.3	0	0.0

Levels of agreement were expressed with a five-point Likert scale that ranged from 1 (Strong disagreement) to 5 (strong agreement)

Asked whether they would agree that the learning material was of nearly identical quality, 60 of 69 participants (87.0%) from Friedberg stated their approval of this claim. Four participants (5.8%) deemed the Athos material superior and no participant considered the JSprit material superior. Five participants (7.2%) refused to answer the question. In the Wetzlar study group, 31 of 32 participants (96.9%) agreed that the learning material was of nearly identical quality. One participant (3.1%) preferred the Athos learning material. No participant opted out of the question.

Regarding the time granted to learn each approach, 58 out of 69 participants (84.1%) from the Friedberg study group would agree that the time for learning the approaches was nearly identical. Six participants (8.7%) felt that they were granted more time for learning JSprit, two participants had the impression that they were granted more time for Athos (2.9%). Three participants (4.3%) did not answer the question. In the Wetzlar study group, 30 out of 32 participants (93.8%) stated, that the time they had for learning both approaches was nearly identical. Two participants (6.3%) felt that they had more time to learn JSprit than they had for Athos. No participant felt that more time for learning Athos was granted and no participant chose not to answer the question.

## Threats to Validity

In this section, we provide a summary of all aspects that we recognized as potential endangerments to the validity of this study. In order to overcome the problem that many experimental studies in software engineering do not use the existing terminology for validity threats (Feldt and Magazinius 2010), we use the terminology presented in Wohlin et al. (2012) for this discussion. A brief overview on the identified threats is presented in Table 10. This table follows the structure used by Kosar et al. (2018). The next sections will provide a detailed discussion of the various threats to the conclusion, internal, construct and external validity of our study. For each identified issue we briefly discuss why it might put the validity of this study at risk and explicate if and how we mitigated the respective threat.

**Table 10:** Summary of the identified threats to the validity of the studies

Validity	Issue	Cause	Addressed
Conclusion	Violated assumption	Unknown distribution of results	Yes
	Reliability	Subjective awarding of points	Yes
	of measures	Secret communication	Yes
	Random heterogeneity	Participants with different prior knowledge and skills	Yes
	Random irrelevancies	Online with no control over physical study environment	No
Internal	Maturation	Occurrence of learning effects	In part
		Occurrence of exhaustion effects	In part
	Instrumentation	Deviating task complexity	Yes
		Deviating training material	Yes
		Deviating time for teaching	Yes
		Formulation of tasks (language)	Yes
		Mistakes in study tasks	Yes
		Biased instruction in the study	No
	Compensation	Unequal incentives	Yes
Construct	Mono-method bias	Effectiveness depends solely on the awarded scores	In part
	Hypothesis guessing	Participants may have worked to the (dis)advantage of Athos	Yes
	Interaction of testing and treatment	Participants may have worked extra carefully to reduce errors	No
External	Interaction of selection and treatment	Subject population consists of students	Yes
	Interaction of setting and treatment	Tasks were presented and to be solved in a survey tool	No
		Usage of simple tasks	No

### Threats to the Conclusion Validity

*Violated assumption* is a threat to the conclusion validity that stems from the selection of inappropriate significance tests, i.e. tests that come with requirements that the obtained data are not guaranteed to meet. In our study, there was no way to be absolutely sure that the scores, the required times (and by extension the PPM) followed a specific distribution (e.g. normal distribution). We addressed this with the application of parameterless tests that do not make any assumptions on the underlying distribution of the obtained samples. However, it is to be noted that this choice bears the potential of introducing *low statistical power* as another threat to conclusion validity due to the fact that paramterless tests are less powerful than their parameterized counterparts in case that the tested data actually follow the assumed distribution.

*Reliability of measures* potentially threatens the validity of the presented study since the results on the effectiveness and efficiency are based on two measures: the score participants were awarded for their answers and the time they spent answering the questions. The latter measure can be objectively taken (but may suffer from *random irrelevancies* discussed in the next paragraph). The score, however, was awarded by the authors of this study and is thus the result of a process that is to some extent affected by subjective human interpretation which may be biased in one way or the other. The attribution of scores could be biased by the researchers towards Athos to achieve the favoured results. Similarly, an overreaching attempt not to favour Athos in the awarded scores might bias the results in favour of JSprit. In order to reduce the influence of subjective marking to a minimum, a fair and objective scoring schema was developed and applied. The scoring schema was developed in a way that the corresponding tasks and the respective steps needed to complete them, were awarded an identical (or at least comparable) amount of points. The scoring schema was especially important for the evolution tasks of the study, in which participants were not limited to a predefined set of option or radio buttons but could enter any model text they deemed fit to solve the task. The schema objectively defined which program parts participants had to add for the solution to be considered correct. It also defined the partial points that each part of the expected code was awarded. While this approach does not completely eliminate the threat to the reliability of the scores, it reduces the risk of bias towards either approach to a minimum.

At this point it is to be mentioned that another threat to the reliability of measures may be posed by the fact that the study was conducted online and the environment thus not entirely controlled by the researchers. It is therefore possible that participants infringed the rule of non-communication between participants. However, we believe that this threat was addressed through the compensation for taking part in the study: participants were only indirectly rewarded for participation (also see below). Participants were explained that due to strict anonymisation there would be no individual reward based on the achieved score. As an incentive for participation it was announced that the tasks of the controlled experiment would serve as an ideal preparation for similar questions that might be part of the final examination (though all training material was provided to all students, i.e. independently of participation). This way, participants had only little reason to break the rules by secretly answering the questions of the study together with a partner. Though we cannot be sure that there was no rule infringement, we are confident that the taken approach served as a mitigation to this threat.

*Random heterogeneity of subjects* can threaten the conclusion validity of the study if participants of a study possess a heterogeneous set of characteristics that have an effect on the observed result. In such groups, there is a chance that the different characteristics of the participants are the actual source for observed differences in the results rather than the applied treatment. One way in which this threat could have occurred in our study would have been through lopsided group assignment. By this we mean the assignment of skilled programmers to the groups in a way so that our preferred outcome was more likely to occur. For example, if we intended to show that Athos was the more effective approach when used as a first approach, results supporting that claim would have been more likely if we assigned experienced and skilled programmers to the Athos first group. This threat, however, was easily eliminated by randomising the group assignment. Even in the face of a randomised group assignment, there would have been a threat from the heterogeneity of subjects if we had mixed participants from Friedberg and Wetzlar and formed groups (Athos first, JSprit first) from the unified pool of participants. In the worst case, all Wetzlar participants together with the most highly skilled participants from Friedberg would have been assigned to one group and all remaining participants to the other. This would certainly have had a significant effect on the between-subjects comparison of both groups. By splitting our study into two studies for either venue, this threat was mitigated. To show that in both studies the groups consisted of similar skilled participants, we asked participants for a self-assessment of their skills as well as their prior knowledge. As was shown in Section 5.2, in both the Friedberg and Wetzlar study, the overall skill and knowledge level in both groups was not skewed in any direction.

*Random irrelevancies in experimental setting* is a threat to the conclusion validity of this study that was not addressed. Since the study was conducted online (see Section 4.3), we had no control over participants’ physical study environment and thus had no way to prevent or track any events that might have had an effect results participants produced. For example, if a person entered the room and talked to a participant, this event is certain to have an effect on the time that a participant requires for the tasks as the disturbances will require the participant to refocus. It may even have an effect on the produced score in case that the unexpected moment of lost concentration causes a participant to provide an incorrect answer. Though we did not explicitly take any measures to ensure that all participants took the study in a controlled environment, there are still characteristics in our study design that might serve as a mitigation to this threat. Firstly, with regard to the between-subjects comparison, the homogeneity of the groups of both studies allows for the assumption that the occurrence of disturbing events is equally distributed among participants of both groups. Secondly, as our study was embedded into the scope of a lecture, participants are likely to have established a routine that made the occurrence of disturbing events during lectures less likely. In addition, we urged participants to not get distracted during the study and focus on answering the questions for both approaches.

### Threats to the Internal Validity

*Maturity* which might result from learning and exhaustion effects poses a major threat to the validity of this study. These effects must be expected to occur since participants first solved a batch of tasks for one approach and after that solve a set of structurally similar tasks with the other approach. It is most likely that participants have learned the structure of a given problem to some extent when confronted with it for a second time. This is likely to result in a higher score for the approach that is applied as the second approach. On the other hand, when participants already answered all questions for a given approach, it is also likely that they might feel mentally exhausted by this point. This most certainly has a negative affect on their concentration and thus most likely negatively affects their score for the second approach. For a pilot study for which no data exists, it is impossible to predict exactly whether (and to what extent) learning effects predominate exhaustion effects or in reverse, exhaustion has a greater effect on the result than knowledge gained from the first time answering the respective question. To avoid these effects entirely, we followed the approach taken by Kosar et al. (2010) and formed two groups (in each study) that applied the two compared approaches in opposite order. Contrary to Kosar et al. (2010), we did not aggregate the results from both sub groups. Instead, we compared the results for the approaches when used as a first approach and also when used as a second approach (see Section 4.11).

*Instrumentation* threats originate from inappropriate material and processes that may confound the observed results. One possible cause that might have threatened the instrumentation of the study would have been a deviation in the complexity of questions (and their respective tasks) which could have seriously compromised the validity of this study. If the questions for one approach had been less difficult than the questions for the other approach, this certainly would have resulted in better scores and reduced the time required for the approach with the simpler questions. As a mitigation, we carefully designed the sections for both approaches so that corresponding questions were of the same structural complexity. This means that corresponding questions had the same number of nodes and edges so that neither was more difficult to comprehend (see Section 4.7). We also ensured that the actions participants were supposed to perform were of comparable complexity, e.g. that they were supposed to add or change the same number of elements in two corresponding questions.

Inferior training material and unequal time for learning the approaches were two more instrumentation threats that we recognised and mitigated: failure to produce training material of comparable quality would have posed a sincere threat to the validity of the study because it most likely would have had a major effect on the obtained results. For this reason, we took care of producing comparable learning aids. We achieved this by defining two generic example VRPTW problems (the one from the introductory case study and a slightly modified version with an additional product and an additional depot). For each approach we created a set of slides that illustrated how to use the respective approach to model the defined example problems. As far as the approaches would allow, the slights showed how to model the problems using the exact same steps (at some points slight deviations were inevitable due to the different structures of Athos and JSprit). Participants also received four pages with example programs (each page with the complete Athos or JSprit program for one of the two example problems).

In the training session, the slides were presented to teach participants how to use the approaches. If one approach had received substantially more teaching time, this would have been another instrumentation threat to the validity of the study. However, we did not define an exact amount of time that was to be spend on Athos and JSprit since both approaches had a different number of slides since JSprit required more lines of code for the example problems. Instead, we defined an upper limit of 90 minutes for the presentation of either approach. Within this time frame, both approaches were presented and all questions that occurred in the scope of the presentation were answered. For both approaches participants were asked whether any part of the presentation required additional discussion or whether there were unanswered questions left. Neither Athos nor JSprit required the full 90 minutes before all questions were answered and participants signalled to have understood the relevant syntactical elements and associated semantics of both approaches. To gain information on whether we were successful in our effort to introduce participants to both approaches in a comparable manner, we asked them in the last section for their feedback on this matter. In both study groups, the vast majority of participants acknowledged that both the time for learning the approaches and the material provided to do so was nearly identical (see Section 5.9).

Another threat to validity concerns the *design of the tasks* participants were supposed to answer in the study. An inappropriate language of the tasks poses a serious threat. Presentation of the tasks in English only (as it is the language in which the study is communicated) in lecture modules for which the official language is German is likely to affect the results as there is a high chance that some participants lack the necessary skills in the language. We thus provided the tasks in both German and English so that participants could switch between the languages at their own discretion.

Even though we took utmost care in setting up the survey and formulating the questions which were also checked by members of the study group, there remained some flaws in the formulation of some of the tasks. This led to participants demanding clarification during the online study, which was provided. However, there is a chance that in some cases participants did not bother or dare to ask for an explanation of an ambiguously or falsely formulated task even though they might not have properly understood it. This may have led to some participants providing answers without fully understanding the question. However, enquiries on the tasks were evenly distributed which is why we consider it unlikely that flaws in the formulations of the tasks have biased the results in favour of any of the two compared approaches.

There was a technical issue with one of the Athos questions^13^ in which 18 checkboxes were presented to participants. The correct answer would have demanded checking five of the boxes but the system would only accept a maximum of three checked boxes. As a remedy, we adapted the marking grid (rating scheme) for this and the corresponding JSprit question so that it was possible to gain the complete score even though not all answers were provided. This way, the originally intended answer key of both the Athos and the corresponding JSprit tasks was modified in favour of both approaches. Though we are convinced that it is unlikely that this approach biased the results towards Athos, it remains a (minor) threat to the validity of the results (for this question). As a general remedy, we are currently conducting a replication of the study in order to validate and verify our results and to further contribute reliable data to researchers in the field of DSL evaluation.

An unaddressed instrumentation threat is posed by the researcher who instructed participants throughout the study: we decided that in all studies one and the same researcher was to introduce participants to the approaches and direct the controlled experiment. This decision addressed the threat that opting for different study instructors would have posed: with different instructors, differences in the results of the groups could have been caused by differing teaching skills of the instructors rather than differences in the approaches or differences in the participants of the study (important for a comparison between the Friedberg and the Wetzlar study). However, the fact that the instructor was involved in the design and implementation of Athos poses a threat to the validity of the study. While every effort was taken to present both approaches in a neutral and objective way, it cannot be guaranteed that the presentation was completely free of any bias, even if only at a subconscious level. These biases of the instructor may have influenced participants in their learning and their answers. It is, however, important to emphasise, that the instructor took care to present both languages in a way that students approached them with an open mind. The threat of subconscious bias during instruction could have been mitigated by having an independent person direct the study. However, this would have posed several other problems: most importantly, no independent person who possessed the necessary knowledge in the domain and the approaches was available as an instructor. An instructor with insufficient knowledge in one or both approaches would have caused an even more severe threat to the study validity.

A last threat to the internal validity was posed by the *compensation* of participants. Since data collection was strictly anonymised, there was no way to directly compensate participants who solved the tasks to the best of their ability. Instead, a question on both approaches was announced to be part of the final exam of both modules in which the study was embedded. In the announcement, it was emphasised that both approaches were of equal importance for the final exam. Neglecting this aspect would have posed a *compensation* threat as participants might have been lead to believe that one of the approaches might be more relevant in the final exam. This would have skewed the effort invested in learning the two different approaches and by extension the achieved scores and required time.

### Threats to the Construct Validity

*Mono-method bias* is a threat to the construct validity of this study that was only partly addressed. In terms of the model activities that participants had to perform, we created a set of questions that covered a wide range of tasks necessary for the specification of VRPTWs so that there is no mono method bias in this regard. However, the fact that the result for participants’ effectiveness is solely based on the score that participants achieved introduces unaddressed mono-method bias into the study. It might be worthwhile to define additional measures for effectiveness in future studies in order to be able to cross check the observed results and thus increase future studies’ validity.

*Hypothesis guessing* threatens the validity of a study if participants conjecture on the purpose of the study and adapt their behaviour in support or opposition of this purpose. We addressed this threat by openly communicating the purpose of the study and asking participants explicitly to not put in extra effort for one of the two languages. We explained, that the most helpful behaviour for us would be to solve the tasks for both approaches with an unprepossessed attitude so that we would be able to gain insight on the particular strengths and weaknesses of our language. We believe, that the vast majority of participants who did not drop out of the study understood the scientific relevance of the study and tried to use both approaches without any prejudice.

*Interaction of testing and treatment* is an unaddressed threat that occurs whenever participants increase their effort due to the knowledge that they are being tested. Even though we asked participants to act as normally as possible, it is evident that there is no way that participants behave the exact same way as they would have behaved when not being in a test situation. The fact that participants were assured that their results were strictly anonymised might slightly mitigate this threat (but it may also lead to a less careful usage of both approaches) as participants had no personal gain from achieving a perfect result. Another possible mitigation to this threat might be that the study was conducted online and thus participants’ physical environment was familiar to them and it did not resemble the typical class room surrounding (again, this might also negatively affect the achieved scores). Thus it is important to note that this threat could not be entirely eliminated.

### Threats to the External Validity

In our study, students enrolled in two different study courses from two different campuses were selected for participation. Though this is a common practice found in the vast majority of controlled experiments in software engineering (Sjoeberg et al. 2005), this type of convenience sampling poses the threat of *interaction of selection and the treatment*. This potential threat occurs if the selected sample population does not appropriately represent the target population so that the generalisation from the sample to the target population is invalid. We addressed this threat by conducting two controlled experiments that represented the two different types of target users of our DSL, i.e. domain experts with little knowledge of software engineering and developers experienced in software development but with limited domain knowledge.

Evidently, the ideal study group is a direct sample of the respective target population. Hence, our sample populations have some inherent limitations with respect to generalisability to our target users. For example, our Friedberg participants who are to be generalised to domain experts, do not possess the same knowledge that domain experts (e.g. fleet dispatchers) have acquired in their job. However, they likely do have a similar (or perhaps slightly superior) level of knowledge in terms of programming languages. Our participants from Wetzlar, on the other hand, might have less programming knowledge than senior software developers, but given that they are all employed by an industrial company that involves them in different software projects, they adequately represent junior software developers. By collection and reporting of data on participants’ programming background, skills and interest, we provided quantitative evidence that allows for an assumption on the degree to which our data can be generalised to the intended target population.

Finally, an unaddressed threat to the external validity of our study can be found in the *interaction of setting and treatment*. This is due to the fact that in our controlled experiment, participants were not allowed to use an IDE. This is a deviation from a real-world industrial working environment were modellers are supported by IDEs that offer mechanisms such as syntax checking and highlighting and code completion features. Though we handed participants two cheat sheets for each approach (in working environments modellers normally are free to use any material that facilitates their work), we explicitly banned the usage of IDEs. This most likely negatively affects the observed scores and times. However, it should not invalidate our results as both approaches were identically affected by this restriction. Another threat that stems from the setting is that the tasks were simplified academic tasks. While they were not trivial, they did not possess the high complexity that would be found in real-world industrial tasks. However, with the simplified tasks, we were able to focus on specific aspects of both languages, so that we believe this design decision to be justified.

## Conclusion

In this study, we gave a brief introduction to Athos and presented a thorough evaluation of the DSL. We gave insight on how the evaluation study was conducted as a controlled experiment in which participants tackled a set of structurally identical tasks with Athos and the Java application library JSprit as a baseline approach. The study was conducted among two study groups with distinctive prior knowledge in terms of software engineering. One study group mostly consisted of students at the beginning of their career as software developers whereas the other comprised only more experienced students enrolled in a co-op study program.

In terms of effectiveness of rather inexperienced users, a within-subjects comparison shows that both the Athos first and the JSprit first group exhibited significantly improved scores with Athos. A between-subjects comparison of the results obtained from inexperienced users shows that when participants used the compared languages as a first approach, Athos users achieved significantly better results than JSprit users. Comparing both languages as a second approach also shows that scores achieved with Athos were higher, however this result is not statistically significant. With regard to efficiency, both within-subjects comparisons (Athos first, JSprit first) show a statistically significant increase in PPM with Athos. The same goes for both between-subjects efficiency comparisons that both show a significantly increased number of PPM with Athos.

These results provide very strong evidence for the claim that Athos is a DSL that benefits language users with only little knowledge in software development. With Athos, these language users can model VRPTWs more effectively and more efficiently than they could do with the GPL alternative. Since participants were second semester students enrolled in a programming related course, special care must be taken when generalising these results. However, we showed that our participants from Friedberg had only little experience in the usage of programming languages. It can thus be assumed that experts from the domain of VRPTWs will similarly benefit from the usage of Athos as a language to model problems from their domain.

For more experienced users, a within-subjects comparison of the exhibited effectiveness reveals that when JSprit was used first and Athos second the scores achieved with Athos are significantly higher than those achieved with JSprit. However, in the other group that used Athos before JSprit, the JSprit results were better, though not at a level of statistical significance. Neither of the two between-subjects comparisons (as first and as second approach) showed a statistically significant advantage for either approach. In terms of observed efficiency among more experienced users, both between-subjects comparisons (as first and as second approach) and one within-subjects comparison (JSprit first) show a statistically significant advantage for our Athos DSL.

These results show that the gains in effectiveness brought about by Athos are less distinctive among more experienced users than they are with programming beginners. This was to be expected since these participants were less familiar with the (descriptive) Athos approach than they were with the imperative Java language. For this reason it is all the more important to stress that one of the tests showed a statistically significant effectiveness increase with Athos. With regard to efficiency the results provide convincing evidence for the claim that even more experienced users become considerably more effective in modelling VRPTWs when using Athos as their modelling language. Since these more experienced participants were software engineering students enrolled in a co-op program, their results can be generalised to professional software developers who support domain experts in the development of traffic and transport simulations.

Not only did Athos enhance the effectiveness and efficiency of users with little programming knowledge and those with advanced experience in software development, it also achieved substantially higher satisfaction levels among both types of language users. The presented results leave no doubt that participants preferred the application of Athos to the usage of the GPL. Though some care must be taken, it seems admissible to generalise these results to domain experts and professional software developers who are likely to also prefer to model VRPTW related problems with our DSL than with the alternative baseline approach. This has important implications since a higher level of satisfaction is likely to positively affect the quality of solutions produced by domain experts and software developers.

With this paper we made the following contributions:

- We provided substantial quantitative evidence in support of the claim that Athos has the potential to increase the effectiveness of program interpretation and creation.
- Our results clearly suggest that especially end users with only limited programming knowledge can greatly benefit from the usage of our DSL. This claim holds merit in terms of achieved effectiveness (score) and efficiency (score in relation to time required).
- Our results provide additional evidence to the claim that the positive effects ensuing from application of a DSL are less distinct for end users with sophisticated knowledge in software engineering.
- The results with regard to the observed user satisfaction provide striking evidence that Athos has the potential to significantly increase the level of felt and expressed contentedness of users. This was shown to be a valid claim for both modellers with little and developers with more advanced programming knowledge.
- With the design of our study we have also expounded the possible effects of the study design on the outcome. Especially when used by experienced programmers, the actual language was less important than possible learning effects. This suggests that when using a *within-subjects comparison* the results should never be interpreted without proper consideration of these effects.

We consider the presented study another valuable contribution to the body of knowledge in the field of DSL evaluation. As our results are generally in line with findings of other researchers in the field, our study adds additional authority to the respective results.

## Supplementary Information

(ZIP 5.81 MB)

## Data Availability

The code for the Athos DSL is made available by the corresponding author upon request.

## Footnotes

1 https://www.eclipse.org/Xtext/

2 https://github.com/Ildevana/Usa-DSL/wiki

3 https://www.eclipse.org/Xtext/

4 https://www.eclipse.org/xtend/

5 https://ccl.northwestern.edu/netlogo/

6 The unit of time is to be defined by the modeller. In the generated NetLogo simulations the amount of time corresponds to simulation ticks.

7 Again, the unit of length is to be defined by the modeller.

8 In this example, there is only one product. In Hoffmann et al. (2020), we give an example where multiple products and demand for multiple products are defined within one program.

9 https://github.com/graphhopper/jsprit

10 Since a group that used Athos first must have used JSprit second, we sometimes use the labels *FbJs (Friedberg, JSprit second)* or *WzJs (Wetzlar, JSprit second)* for these groups.

11 Depending on context, sometimes these groups are referred to as *FbAs (Friedberg, Athos second)* and *WzAs (Wetzlar, Athos second)*.

12 This is a logical and not an exclusive or.

13 Question Q01ATNW
