# Supplementary material for: An empirical evaluation of a novel domain-specific language – modelling vehicle routing problems with Athos
Source: Empir Softw Eng. 2022 Sep 23;27(7):180. doi: 10.1007/s10664-022-10210-w (PMC9510508; doi:10.1007/s10664-022-10210-w)
Supplement: Supplementary file 1 — (ZIP 5.81 MB) [file 10664_2022_10210_MOESM1_ESM.zip › TitlePage.pdf]

Noname manuscript No.
(will be inserted by the editor)

An Empirical Evaluation of a Novel Domain-Specific Language – Modelling Vehicle Routing Problems with Athos

Benjamin Hoffmann · Neil Urquhart · Kevin Chalmers · Michael Guckert

Received: date / Accepted: date

Abstract Domain-specific languages (DSLs) are a popular approach among software engineers who demand for a tailored development interface. A DSL-based approach allows to encapsulate the intricacies of the target platform in transformations that turn DSL models into executable software code. Often, DSLs are even claimed to reduce development complexity to a level that allows them to be successfully applied by domain-experts with limited programming knowledge. Recent research has produced some scientifically backed insights on the benefits and limitations of DSLs. Further empirical studies are required to build a sufficient body of knowledge from which support for different claims related to DSLs can be derived. In this research study, we adopt current DSL evaluation approaches to investigate potential gains, in terms of effectiveness and efficiency, through the application of our DSL *Athos*, a language developed for the domain of traffic and transportation simulation and optimisation. We compare Athos to the alternative of using an application library defined within a general-purpose language (GPL). We specify two sets of structurally identical tasks from the domain of vehicle routing problems and asked study groups with differing levels of programming knowledge to solve the tasks with the two approaches. The results show that inexperienced participants achieved considerable gains in effectiveness and efficiency with the usage of Athos

Benjamin Hoffmann ✉
Technische Hochschule Mittelhessen, Kompetenzzentrum für Informationstechnologie
Friedberg, Germany
E-mail: benjamin.hoffmann@mnd.thm.de

Neil Urquhart
Edinburgh Napier University, School of Computing
Edinburgh, Scotland
E-mail: N.Urquhart@napier.ac.uk

Kevin Chalmers
University of Roehampton, School of Arts
London, England E-mail: Kevin.Chalmers@roehampton.ac.uk

Michael Guckert
Technische Hochschule Mittelhessen, Kompetenzzentrum für Informationstechnologie
Friedberg, Germany
E-mail: michael.guckert@mnd.thm.de
